# Convergent Evolution of Calcineurin Pathway Roles in Thermotolerance and Virulence in *Candida glabrata*

**DOI:** 10.1534/g3.112.002279

**Published:** 2012-06-01

**Authors:** Ying-Lien Chen, Jay H. Konieczka, Deborah J. Springer, Samantha E. Bowen, Jing Zhang, Fitz Gerald S. Silao, Alice Alma C. Bungay, Ursela G. Bigol, Marilou G. Nicolas, Soman N. Abraham, Dawn A. Thompson, Aviv Regev, Joseph Heitman

**Affiliations:** * Department of Molecular Genetics and Microbiology; ‡‡ Pathology, and; §§ Immunology, Duke University Medical Center, Durham, North Carolina 27710; † The Broad Institute of MIT & Harvard, Cambridge, Massachusetts 02142; †† Howard Hughes Medical Institute, Department of Biology, Massachusetts Institute of Technology, Cambridge, Massachusetts 02142; ‡ Department of Chemistry, Duke University, Durham, North Carolina 27710; § Department of Microbiology and Parasitology, University of Perpetual Help–Dr. Jose G. Tamayo Medical University, Biñan, Laguna, 4024 Philippines; ** National Institutes of Health, University of the Philippines, Manila, Philippines; †† Environment and Biotechnology Division, Industrial Technology Development Institute, Department of Science and Technology, Bicutan, Taguig City, 1631 Philippines

**Keywords:** phosphatase, calcium, calmodulin, Crz1, Rcn1, Rcn2, thermotolerance, cell wall integrity, ER stress, drug tolerance, pH homeostasis, urinary tract infection, ocular infection, virulence

## Abstract

*Candida glabrata* is an emerging human fungal pathogen that is frequently drug tolerant, resulting in difficulties in treatment and a higher mortality in immunocompromised patients. The calcium-activated protein phosphatase calcineurin plays critical roles in controlling drug tolerance, hyphal growth, and virulence in diverse fungal pathogens via distinct mechanisms involving survival in serum or growth at host temperature (37° and higher). Here, we comprehensively studied the calcineurin signaling cascade in *C. glabrata* and found novel and uncharacterized functions of calcineurin and its downstream target Crz1 in governing thermotolerance, intracellular architecture, and pathogenesis in murine ocular, urinary tract, and systemic infections. This represents a second independent origin of a role for calcineurin in thermotolerant growth of a major human fungal pathogen, distinct from that which arose independently in *Cryptococcus neoformans*. Calcineurin also promotes survival of *C. glabrata* in serum via mechanisms distinct from *C. albicans* and thereby enables establishment of tissue colonization in a murine systemic infection model. To understand calcineurin signaling in detail, we performed global transcript profiling analysis and identified calcineurin- and Crz1-dependent genes in *C. glabrata* involved in cell wall biosynthesis, heat shock responses, and calcineurin function. Regulators of calcineurin (RCN) are a novel family of calcineurin modifiers, and two members of this family were identified in *C. glabrata*: Rcn1 and Rcn2. Our studies demonstrate that Rcn2 expression is controlled by calcineurin and Crz1 to function as a feedback inhibitor of calcineurin in a circuit required for calcium tolerance in *C. glabrata*. In contrast, the calcineurin regulator Rcn1 activates calcineurin signaling. Interestingly, neither Rcn1 nor Rcn2 is required for virulence in a murine systemic infection model. Taken together, our findings show that calcineurin signaling plays critical roles in thermotolerance and virulence, and that Rcn1 and Rcn2 have opposing functions in controlling calcineurin signaling in *C. glabrata*.

*Candida glabrata* is an emerging human fungal pathogen. Most strains have reduced antifungal drug susceptibility, thus making treatment challenging (Lagrotteria *et al.* 2007; Pfaller *et al.* 2011a). The poor susceptibility of *C. glabrata* to antifungal drugs contributes to the high mortality rate (∼50%) associated with *C. glabrata* candidemia (Klevay *et al.* 2009). Resistance to echinocandins and azoles was most prevalent among nosocomial bloodstream infection (BSI) isolates of *C. glabrata* compared with other *Candida* species (Pfaller *et al.* 2011a). Although *C. albicans* accounts for 48% of *Candida* BSIs, *C. glabrata* ranked second, accounting for 18% of infections across multiple geographic regions, including Asia-Pacific, Europe, Latin America, and North America (Pfaller *et al.* 2011b). Besides BSI, *Candida* species also cause urinary tract infections (UTI). Fungal UTIs are often caused by *Candida* species, which account for ∼10% of all nosocomial UTIs related to indwelling catheters, with *C. glabrata* accounting for approximately 15% of all *Candida* isolates (Kauffman *et al.* 2000; Lundstrom and Sobel 2001). Surprisingly, a study performed by Phillips and Karlowicz (1997) showed that 42% of hospital-acquired urinary tract infections in neonatal intensive care units were caused by *Candida* species. For *Candida* ocular infections, *C. glabrata* was previously rarely seen; however, several recent studies report that *C. glabrata* infects human corneas, especially in patients undergoing keratoplasty (Caldwell *et al.* 2009; Djalilian *et al.* 2001; Kitzmann *et al.* 2009; Lee *et al.* 2011; Tappeiner *et al.* 2009). The pathogenic mechanisms by which *C. glabrata* causes ocular infections have not been reported.

Calcineurin is a calcium/calmodulin-dependent serine/threonine-specific protein phosphatase that comprises a catalytic A (Cna1) and a regulatory B calcium-binding subunit (Cnb1). Upon stimulation with calcium, calmodulin associates with the calcineurin AB heterodimer, stimulating phosphatase activity and converting signals to various outputs by dephosphorylating its downstream targets. Upon dephosphorylation by activated calcineurin, the transcription factor Crz1 in fungi or nuclear factor of activated T cells (NFAT) in mammals migrates to the nucleus to regulate gene expression. Because active calcineurin is an AB heterodimer, the loss of Cnb1 often results in destabilization of Cna1 (Chen *et al.* 2010a). Calcineurin is essential for growth at elevated temperatures in the human fungal pathogen *Cryptococcus neoformans* (Odom *et al.* 1997) and the protozoan parasite *Leishmania major* (Naderer *et al.* 2011), but it has not been demonstrated to have a role in controlling thermotolerance of ascomycetes, including *Candida* species. The intracellular architecture defects of *C. neoformans* and *L. major* calcineurin mutants upon thermal stress have not been investigated. In *C. glabrata*, calcineurin and Crz1 have been demonstrated to play functions in antifungal drug tolerance, cell wall integrity, and virulence in a murine systemic infection model (Miyazaki *et al.* 2010). However, the mechanism for *C. glabrata* calcineurin and Crz1 requirement in serum survival and virulence in other murine infection models remains elusive.

The calcineurin- and/or Crz1-regulated transcriptome has been investigated in *Saccharomyces cerevisiae*, *C. albicans*, *Aspergillus fumigatus*, and the rice blast fungal pathogen *Magnaporthe oryzae* (Karababa *et al.* 2006; Kim *et al.* 2010; Malavazi *et al.* 2009; Yoshimoto *et al.* 2002). The regulation pattern of the transcriptome is diverged among the four species. Because *C. glabrata* calcineurin and *crz1* mutants exhibited novel phenotypes distinct from these species in terms of hypersensitivity to thermal stress, ER stress, and antifungal drugs, microarray analyses to explore the targets of calcineurin and Crz1 in *C. glabrata* will extend our understanding of calcineurin signaling.

Regulators of calcineurin (RCN) are a novel family of calcineurin regulators found in eukaryotic cells. In *S. cerevisiae*, two regulators of calcineurin (Rcn1 and Rcn2) have been demonstrated to control calcineurin signaling. In *C. albicans*, the function of Rcn1 has been associated with calcineurin signaling (Reedy *et al.* 2009). In *C. glabrata*, the functions of Rcn1 and Rcn2 in controlling calcineurin signaling were recently reported (Miyazaki *et al.* 2011a). *C. glabrata* Rcn1 displayed both stimulatory and inhibitory effects, whereas Rcn2 showed only inhibitory activities on calcineurin signaling (Miyazaki *et al.* 2011a). Interestingly, Rcn1 is required for micafungin and fluconazole tolerance in addition to ER stress induced by tunicamycin (Miyazaki *et al.* 2011a). However, the roles of Rcn1 and Rcn2 in temperature sensitivity, pH response, and virulence remain unclear. In mammals, the homolog of *S. cerevisiae* Rcn1 is named DSCR1/Rcan1/calcipressin and is overexpressed in the brain of patients with Down’s syndrome (Rothermel *et al.* 2003). In addition, Baek *et al.* (2009) demonstrated that DSCR1, a negative regulator of calcineurin located on chromosome 21 and thus present in three copies in patients with Down’s syndrome, has antiangiogenic activity and inhibits tumor formation.

In this study, we comprehensively studied the roles of the calcineurin signaling cascade in stress responses, antifungal drug tolerance, and pathogenesis of *C. glabrata*. We demonstrated that *C. glabrata* calcineurin (Cna1 and Cnb1) and/or Crz1 are required for thermotolerance, ER stress, pH homeostasis, and virulence in murine ocular, urinary tract, and systemic infection models. In addition, global transcript profiling analysis revealed 30 calcineurin and Crz1-activated targets, which explain, at least in part, the requirement of calcineurin signaling in cell wall integrity, thermotolerance, and pathogenesis. Furthermore, we demonstrated that a novel regulator of calcineurin 2 (Rcn2) is a downstream target of calcineurin and Crz1 and acts as a feedback inhibitor of calcineurin signaling in response to calcium, whereas Rcn1 functions as a positive regulator of calcineurin signaling.

## Materials and Methods

### Ethics statement

Animals studies conducted in the Division of Laboratory Animal Resources (DLAR) facilities at Duke University Medical Center (DUMC) were handled with good practice as defined by the United States Animal Welfare Act and in full compliance with the guidelines of the DUMC Institutional Animal Care and Use Committee (IACUC). The murine systemic and urinary tract infection models were reviewed and approved by the DUMC IACUC under protocol numbers A238-09-08 and A219-08-08, respectively.

Murine ocular infection studies conducted at the animal house facility of the Department of Microbiology and Parasitology, University of Perpetual Help–Dr. Jose G. Tamayo Medical University (UPH-DJGTMU) were performed in accordance with the ARVO Statement for the Use of Animals in Ophthalmic and Vision Research, the United States Animal Welfare Act, and the Republic of the Philippines Animal Welfare Act of 1998 (RA No. 8485), and in full conformity with the guidelines set forth in the UPH-DJGTMU research manual. The protocol was formally approved by the UPH-DJGTMU institutional review board after review by the Regional Institute for Tropical Medicine Institutional Animal Care and Use Committee (RITM-IACUC) under research protocol No. 010. All animal infection experiments were conducted by properly trained personnel, including licensed veterinarians.

### Yeast strains, media, and chemicals

Yeast strains used in this study are listed in Table 1. YPD (1% yeast extract, 2% peptone, 2% glucose) liquid medium and agar (2%) and SC (6.7 g yeast nitrogen base without amino acids, 1 × amino acid stock, 20 g glucose, 20 g agar in 1 liter) media were used in this study. YPD medium containing 100 µg/ml nourseothricin was used to select transformants. YPD medium containing 150 mM HEPES buffered at pH 2, 5, 8, or 9 was used to study pH tolerance. FK506 (Astellas Pharma Inc.), cyclosporin A (CsA; LC Laboratories), sodium dodecyl sulfate (SDS; Fisher), fetal bovine serum (Invitrogen), calcofluor white (CFW, fluorescent brightener 28; Sigma), Congo red (Sigma), fluconazole (Bedford Laboratories), posaconazole (Sequoia Research Products Ltd.), ketoconazole (Sigma), voriconazole (Sigma), caspofungin (Merck), micafungin (Astellas Pharma Inc.), anidulafungin (Pfizer Inc.), tunicamycin (Sigma), and dithiothreitol (DTT; Sigma) were added to the media at the concentrations indicated.

**Table 1:** *Candida glabrata* and *Cryptococcus neoformans* strains

Strain	Genotype	Parent	Reference
*C. glabrata*		
CBS138	Prototrophic wild-type	Clinical isolate	Dujon *et al.* (2004)
YC67*^a^*	*cna1*Δ::*SAT1-FLP*	CBS138	This study
YC98*^a^*	*cna1*Δ:: *SAT1-FLP*	CBS138	This study
YC191*^b^*	*cnb1*Δ::*SAT1-FLP*	CBS138	This study
YC193*^b^*	*cnb1*Δ::*SAT1-FLP*	CBS138	This study
YC267*^c^*	*crz1*Δ::*SAT1-FLP*	CBS138	This study
YC182*^c^*	*crz1*Δ::*SAT1-FLP*	CBS138	This study
YC553*^d^*	*rcn1*Δ::*SAT1-FLP*	CBS138	This study
YC556*^d^*	*rcn1*Δ::*SAT1-FLP*	CBS138	This study
YC525*^e^*	*rcn2*Δ::*SAT1-FLP*	CBS138	This study
YC531*^e^*	*rcn2*Δ::*SAT1-FLP*	CBS138	This study
YC581	*rcn2*Δ::*FRT*	YC531	This study
YC594	*rcn2*Δ::*FRT rcn1*Δ::*SAT1-FLP*	YC581	This study
YC111	*cna1*Δ::*FRT*	YC98	This study
YC535*^f^*	*cna1*Δ::*FRT rcn2*Δ::*SAT1-FLP*	YC111	This study
YC549*^f^*	*cna1*Δ::*FRT rcn2*Δ::*SAT1-FLP*	YC111	This study
YC504	*cnb1*Δ::*FRT*	YC193	This study
YC599*^g^*	*cnb1*Δ::*FRT rcn2*Δ::*SAT1-FLP*	YC504	This study
YC601*^g^*	*cnb1*Δ::*FRT rcn2*Δ::*SAT1-FLP*	YC504	This study
YC502	*crz1*Δ::*FRT*	YC182	This study
YC532*^h^*	*crz1*Δ::*FRT rcn2*Δ::*SAT1-FLP*	YC502	This study
YC534*^h^*	*crz1*Δ::*FRT rcn2*Δ::*SAT1-FLP*	YC502	This study
*C. neoformans*			
H99	Prototrophic wild-type	Clinical isolate	Perfect *et al.* (1993)
KK1	*cna1*Δ::*NAT*	H99	Kojima *et al.* (2006)

a,b,c,d,e,f,g,h Two independently derived *cna1*, *cnb1*, *crz1*, *rcn1*, *rcn2*, *cna1 rcn2*, *cnb1 rcn2*, or *crz1 rcn2* mutants, respectively.

### Identification of *C. glabrata* gene orthologs

The *C. glabrata* orthologs of the genes encoding *C. albicans* calcineurin subunits Cna1 and Cnb1 and the calcineurin target Crz1 were identified by reciprocal BLAST searches between the two species, with the reciprocal best BLAST hit orthologs in *C. glabrata* being the *CNA1* (*CAGL0L11110g*), *CNB1* (*CAGL0L00605g*), and *CRZ1* (*CAGL0M06831g*) genes. *C. glabrata* YPS5 (CAGL0E01771g, putative aspartyl protease) and *RCN2* (*CAGL0J04158g*, regulator of calcineurin 2) were identified by reciprocal BLAST searches using the *S. cerevisiae* genome and genes. The *C. glabrata* ortholog of *C. albicans* RCN1 (regulator of calcineurin 1) was identified by reciprocal BLAST searches between *C. glabrata* and *C. albicans*, identifying the reciprocal best BLAST hit as *C. glabrata* *RCN1* (*CAGL0E06248*).

### Gene disruptions in *C. glabrata*

All deletion strains were generated from the prototrophic CBS138 (ATCC2001) background using the *SAT1* flipper (Reuss *et al.* 2004). All primers used in strain construction are listed in Table S1. For the *CNA1* gene disruption, approximately 1 kb of the 5′ (amplified with primers JC49/JC50, Table S1) and 3′ (amplified with primers JC51/JC52) noncoding regions (NCR) of the *CNA1* ORF were PCR-amplified from genomic DNA of the wild-type strain CBS138. The 4.2 kb *SAT1* flipper sequence was amplified from plasmid pSFS2A (Reuss *et al.* 2004) with primers JC17/JC18. The three PCR products were treated with ExoSAP-IT (USB Corp.) to remove contaminating primers and dNTPs and then combined in a 1:3:1 molar ratio (5′ *CNA1*^NCR^: *SAT1* flipper: 3′ *CNA1*^NCR^) to generate the disruption allele by overlap PCR using flanking primers JC49/JC52, resulting in an ∼6.2 kb 5′*CNA1*^NCR^-*SAT1* flipper-3′*CNA1*^NCR^ *CNA1* disruption allele. The *CNA1* gene was disrupted in the wild-type strain CBS138 by transformation with 0.2∼1.0 µg of gel-purified disruption cassette DNA extracted with the Frozen-EZ Yeast Transformation Kit (Zymo Research). Two independent nourseothricin-resistant *cna1* mutants (YC67 and YC98, Table 1) were obtained from two separate transformations.

A similar approach was employed to disrupt the *CNB1* and *CRZ1* genes, with ∼1 kb of the 5′ and 3′ noncoding regions used for homologous recombination. To generate the ∼6 kb *cnb1* disruption allele, the overlap PCR DNA products 5′ *CNB1*^NCR^ (amplified with primers JC134/JC135), *SAT1* flipper (amplified with primers JC17/JC18), and 3′ *CNB1*^NCR^ (amplified with primers JC136/JC137) were mixed in a 1:3:1 molar ratio and amplified with primers JC138/JC139 (∼100 bp closer to the *CNB1* ORF compared with JC134/JC137, respectively, reserving primers JC134/JC137 for further integration confirmation). Two independent nourseothricin-resistant *cnb1* mutants (YC191 and YC193, Table 1) derived from two separate transformations were obtained. To generate the ∼6 kb *crz1* disruption allele, 5′ *CRZ1*^NCR^ (amplified with primers JC142/JC143), *SAT1* flipper (amplified with primers JC17/JC18), and 3′ *CRZ1*^NCR^ (amplified with primers JC144/JC145) were combined and amplified with primers JC146/JC147 (∼100 bp closer to the *CRZ1* ORF compared with JC142/JC145, respectively). Two independent nourseothricin-resistant *crz1* mutants (YC267 and YC182, Table 1) derived from two separate transformations were obtained.

To disrupt the *RCN1* gene, 5′ *RCN1*^NCR^ and 3′ *RCN1*^NCR^ were amplified with primers JC459/JC460 and JC461/JC462, respectively. JC463/JC464 primers were used to amplify the *RCN1* disruption allele via overlap PCR including three fragments: 5′ *RCN1*^NCR^, *SAT1* flipper, and 3′ *RCN1*^NCR^. Two independent *rcn1* mutants (YC553 and YC556) were obtained. To disrupt the *RCN2* gene, 5′ *RCN2*^NCR^ and 3′ *RCN2*^NCR^ were amplified with primers JC441/JC442 and JC443/JC444, respectively. JC445/JC446 primers were used to amplify the *RCN2* disruption allele via overlap PCR including three fragments: 5′ *RCN2*^NCR^, *SAT1* flipper, and 3′ *RCN2*^NCR^. Two independent *rcn2* mutants (YC525 and YC531) were obtained.

To obtain the *rcn1 rcn2* double mutant, we disrupted the *RCN1* gene in the *rcn2* mutant background (YC581, the *SAT1* flipper was removed via culturing YC531 in YPD medium and replicating onto nourseothricin-containing medium to confirm the loss of *SAT1* flipper). To obtain the *cna1* *rcn2* (YC535 and YC549), *cnb1 rcn2* (YC599 and YC601), and *crz1 rcn2* (YC532 and YC534) double mutants, we disrupted the *RCN2* gene in *SAT1* flipper-free (methods described above) *cna1* (YC111), *cnb1* (YC504), and *crz1* (YC502) mutants, respectively.

### Transmission electron microscopy

Transmission electron microscopy (TEM) of *C. glabrata* was accomplished as follows. Cells were grown overnight in YPD at 24° and washed twice with dH_2_O. Then 0.1 OD_600_ of cells (in 100 µl) was spread on YPD agar plates and incubated for 24 hr at 24°, 37°, and 40°. Cells were collected from agar plates by washing in 0.2 M sodium cacodylate buffer (pH = 6.8), collected by centrifugation (∼4,000 rpm, 3 min in a table-top centrifuge), resuspended in 2% glutaraldehyde plus 0.05% malachite green oxalate in 0.1 M sodium cacodylate buffer, and incubated at 4° for 2 days. Fixed cells were collected by centrifugation, resuspended, washed with 0.1 M sodium cacodylate buffer, centrifuged, supernatant removed, and post-fixed with 0.8% K_3_Fe(CN)_6_, 1% OsO_4_, 0.1 M sodium cacodylate for 2 hr at room temperature. The cells were washed twice with 0.1 M sodium cacodylate buffer and stained with 1% tannic acid for 1 hr at room temperature. Then cells were washed with 0.1% sodium cacodylate buffer for 5 min followed by two washes in dH_2_0 for 5–10 min each and stained with 1% uranyl acetate in water overnight at 4°. Samples were washed in molecular-grade distilled water, embedded in an agarose pellet, and prepared for embedding. The cured peg was trimmed, sectioned, and mounted on copper grids. Grids were post-stained prior to viewing. Sections were viewed and imaged with a Philips/FEI CM 12 Transmission EM (FEI Company, Hillsboro, OR) with Advanced Microscopy Techniques Corp. (AMT) 2K × 2K digital camera (Danvers, MA) at the Duke University Department of Pathology.

### Fluorescence microscopy

Cells were grown overnight in YPD at 24° and washed twice with dH_2_O. Then 0.1 OD_600_ of cells (in 100 µl) was spread on YPD agar plates and incubated for 24 hr at 24° and 37°. The Alexa Fluor 488 phalloidin (Cat #A12379; Life Technologies) staining of actin was performed based on the protocol described by Adams and Pringle (1991) with minor modifications. In brief, cells were spun and resuspended in 3.7% (vol/vol) formaldehyde in PBS for 1 hr, washed, and resuspended in PBS (100 µl). Cells were then stained with 20 µl of 6.6 µM (or 0.2 U/µl) phalloidin stock for 1 hr in the dark. Stained cells were washed five times with PBS, resuspended in 100 µl PBS in the absence of mounting medium, and observed using the standard green fluorescence filter set.

### Time-kill curve for strains exposed to fluconazole, micafungin, or serum

Cells were grown overnight at 24°, washed twice with dH_2_O, counted with a hemocytometer, and then 5 × 10^6^ cells were added to 5 ml of fresh YPD medium ± fluconazole/micafungin or 5 ml of 100% fetal bovine serum to achieve 10^6^ cells/ml. Cells were cultured at 24° with shaking at 250 rpm. The cells surviving after 0, 3, 6, 9, and 24 hr were serially diluted onto YPD medium, and CFUs were counted after 48 hr of incubation at 24°. The experiments were performed in triplicate, and data were plotted using Prism 5.03.

### Culture growth, harvesting, and total RNA extractions for microarray experiments

Strains were grown overnight at 24°, washed twice with dH_2_O, diluted to 0.2 OD_600_/ml in YPD, and incubated for 3 hr at 24°. For wild-type strains, cells in log-phase were diluted to 0.2 OD_600_/ml (10 ml) in YPD in the presence or absence of FK506 (1 µg/ml) while *cna1* (YC98) and *crz1* (YC182) mutants were diluted to 0.2 OD_600_/ml (10 ml) in YPD. Following 3 hr of incubation at 37° with shaking at 250 rpm, the 10 ml cultures were immediately added to 15 ml methanol (60%) chilled in a dry ice-ethanol bath to stop cellular processes and RNase activity. Cells were pelleted at 3000 rpm at −4°, flash frozen with liquid N_2_, and stored at −80° prior to total RNA extraction. The total RNAs were extracted using the RNeasy Mini Kit (Qiagen). RNA quality was assessed with the RNA 6000 Nano Kit of the Agilent Bioanalyzer 2100 to ensure RNA Integrity Number (RIN) scores ≥ 7.

### Probe preparation, microarray hybridization, and data analysis

Total RNA samples were reverse-transcribed to cDNA and labeled with either Cy3 or Cy5, according to a modification of the protocol described by Wapinski *et al.* (2010). We employed a wheel hybridization design to compare all samples to each other with dye-swaps, for four comparisons per sample on 10 microarrays per biological replicate. The entire hybridization scheme was repeated for two biological replicates. After hybridization and washing per the manufacturer’s instructions, arrays were scanned using an Agilent scanner and analyzed with Agilent’s Feature Extraction software V10.5.1. The median intensities of probes corresponding to a specific ORF were used to estimate the expression values for each gene. We then fit linear models to these gene expression data using the limma library of the R Bioconductor Package (Gentleman *et al.* 2005), which computes fold change, indicates the direction and quantity of the differential gene expression between the samples, and provides summary statistics, including T- and B-statistics and an adjusted *P* value that takes into account the false discovery rate. For each comparison in each study, Q-values were computed using the R package. Microarray results have been deposited at NCBI Gene Expression Omnibus (http://www.ncbi.nlm.nih.gov/geo/query/acc.cgi?acc=GSE31167).

### Quantitative determination of expression by real-time RT-PCR

DNase I (Turbo DNA-free; Ambion) was used to eliminate genomic DNA contamination. One microgram of DNA-free total RNAs was reverse-transcribed to cDNA by the Affinity Script qPCR cDNA Synthesis Kit (Agilent). PCR reactions of 25 µl included 10 ng cDNA (in 10 µl), 12.5 µl of 2× qPCR master mix (Brilliant SYBR Green Kit; Agilent), 0.5 µl of 5 µM forward primer, 0.5 µl of 5 µM reverse primer, 1.125 µl of nuclease-free H_2_O, and 0.375 µl of ROX dye. Quantitative PCR conditions were the following: 95°/10 min (denaturation); 95°/15 sec, 60°/1 min (40×, cycling stage); 95°/15 sec, 60°/1 min, 95°/15 sec (melting curve). Primers for probes were designed using Primer3 (http://frodo.wi.mit.edu/primer3/) and are listed in Table S1. The ABI PRISM 7900HT machine and StepOne v2.1 (Applied Biosystems) were used to determine ΔΔCt and relative quantity (RQ). The bar graphs of *ACT1* normalized RQ compared with the wild-type (CBS138) were created with Prism 5.03.

### Mouse studies

#### Urinary tract infection model:

Six- to eight-week-old female C3H/HeJ mice (stock #000659, n = 5 for each group) and C3H/HeOuJ (stock #000635, n = 8 for the group) from the Jackson Laboratory were used in this study. *C. glabrata* strains were grown in 5 ml SD + CAA medium (6.7 g yeast nitrogen base minus amino acids, 6 g casamino acids, 20 g glucose in 1 L dH_2_O) overnight at 24°. Cultures were washed twice with 10 ml of phosphate buffered saline (PBS) and diluted to yield an infection inocula of 10^9^ cells/ml after hemocytometer counting. Prior to infection, mice were anesthetized with i.p. injection of 100 µl of 5-fold diluted pentobarbital (final concentration of 10 mg/ml in PBS). Thirty microliters (3 × 10^7^ cells) were used to infect mice via the urinary tract for 15 sec aided by polyethylene catheter tubing (BD catalog #427400) and syringe (BD catalog #309659) with 30 G × 1/2 needle (BD catalog #305106). Bladder and kidney tissue samples were harvested on day 7 and homogenized with 5 ml PBS for 5 s at 17,500 rpm (for kidneys) or at 24,000 rpm (for bladder) (Power Gen 500; Fisher Scientific). Tissue homogenates were serially diluted and 100 µl was plated onto YPD solid medium. The plates were incubated at 24° for 72 hr to determine CFUs per organ. Appropriate dilutions of the inocula were plated onto YPD at 24° for 48 hr to confirm cell viability. All experimental procedures were carried out according to NIH guidelines and Duke IACUC protocols for the ethical treatment of animals.

#### Murine systemic infection model:

Five- to six-week-old male CD1 mice from the Jackson Laboratory (n = 10 for each group) were utilized in this study. *C. glabrata* strains were grown in 5 ml liquid YPD medium overnight at 24°. Cultures were washed twice with 10 ml of phosphate buffered saline (PBS), and the cells were then resuspended in 2 ml of PBS. Cells were counted with a hemocytometer and resuspended in an appropriate volume of PBS to obtain an infection inocula concentration of 2 × 10^8^ cells/ml. Two hundred microliters (4 × 10^7^ cells) were used to infect mice by lateral tail vein injection. Appropriate dilutions of the cells were plated onto YPD solid medium and incubated at 24° for 48 hr to confirm cell viability. *C. glabrata*–infected mice were sacrificed and dissected on day 7 post-infection. The kidney and spleen tissues were removed, weighed, transferred to a 15 ml Falcon tube filled with 5 ml PBS, and homogenized for 5 s at 19,000 rpm (IKA T25; Cole-Parmer). Tissue homogenates were serially diluted, and 100 µl was plated onto YPD solid medium. The plates were incubated at 24° for 48 hr to determine CFUs per gram of organ. The identity of organ-recovered colonies was confirmed by PCR. All experimental procedures were carried out according to NIH guidelines and Duke IACUC protocols for the ethical treatment of animals.

#### Murine ocular infection model:

*Candida* strains were grown in YPD overnight at 25°. Cultures were washed three times with sterile PBS (pH 7.4). Cells were diluted to a concentration of 10^6^ CFU/5 μl. The concentration was determined by using the spectrophotometer optical density reading at a wavelength of 600 nm and multiplying it by a conversion factor of 1 OD_600_, equivalent to 3 × 10^7^ cells/ml. Inoculum concentration was verified by plating on YPD for 48 hr at 25°.

For murine ocular infection, six- to eight-week-old outbred ICR mice (20–28 g; Research Institute for Tropical Medicine, Alabang, Philippines) were used in the experiment in accordance with the ARVO Statement for the Use of Animals in Ophthalmic and Vision Research. The previously described keratomycosis protocol (Chen *et al.* 2011) was used for *Candida* infection with minor modifications and was approved by the University of Perpetual Help Institutional Review Board. Before the infection procedure, mice were anesthetized by intravenous injection of Zoletil 50 (10–15 mg/kg body weight; Virac, Australia) followed by topical application of proparacaine hydrochloride ophthalmic solution (Alcaine; Alcon-Couvreur, Belgium) to the eyes. Once animals were anesthetized, the right eye was superficially scarified in a grid-pattern by a sterile 25-gauge hypodermic needle, and 5 μl of *Candida* solution (10^6^ CFU) was placed into each eye. The inoculum was distributed uniformly by rubbing the eye for few seconds with the eyelid. A mock-infection experiment was performed using sterile PBS as a control. Disease severity of fungal keratitis was assessed for 8 days with the aid of a dissecting microscope. In this procedure, corneal involvement was assessed and scored according to three parameters: (i) area of opacity, (ii) density of opacity, and (iii) surface regularity. A grade of 0 to 4 was assigned based on each of these criteria to yield a maximum score of 12.

### Statistical analysis

Statistical analysis was conducted using Prism 5.03 software (GraphPad, La Jolla, CA). The significance of differences in fungal burden was determined using one-way ANOVA and Dunnett’s multiple comparison tests. For optimal serum growth and real-time RT-PCR, the significance of differences was determined by one-way ANOVA and Bonferroni’s multiple comparison tests. For shrunken cell percentage and murine ocular infection, the Student unpaired *t*-test was used to determine significance. *P* < 0.05 was considered significant.

## Results and Discussion

### The calcineurin pathway plays a critical role in thermotolerance in *C. glabrata*

**Figure 1:**
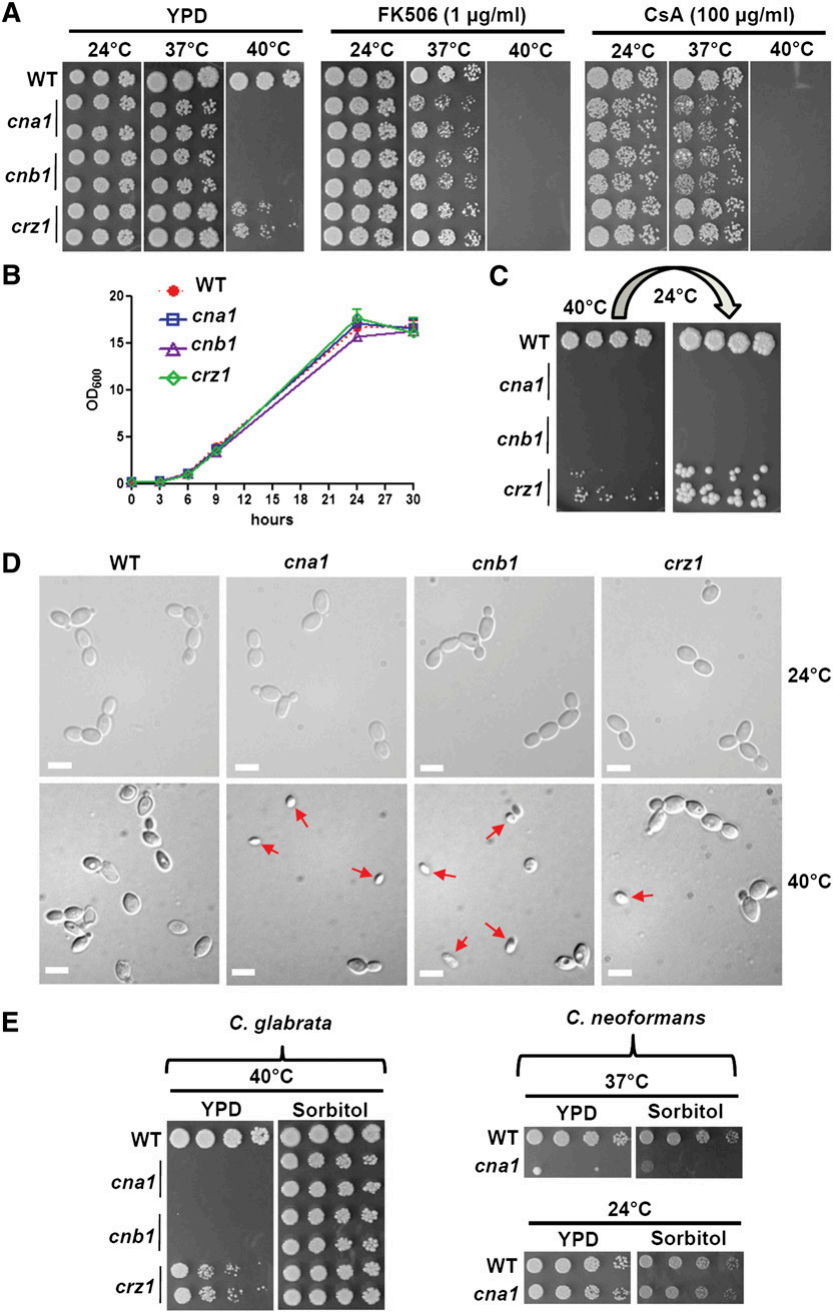
Calcineurin pathway is required for thermotolerance in *C. glabrata*. (A) Pharmacological inhibition of calcineurin phenocopies genetic deletion of calcineurin. Cells were grown overnight in YPD at 24°, 5-fold serially diluted, and spotted onto YPD medium containing FK506 or cyclosporin A (CsA), and incubated at the indicated temperatures for 48 hr. Strains tested were wild-type (CBS138), *cna1* mutants (YC67 and YC98), *cnb1* mutants (YC191 and YC193), and *crz1* mutants (YC267 and YC182). (B) The growth kinetics of *C. glabrata* wild-type and mutant strains at 24°. Cells were grown overnight at 24°, washed twice with dH_2_O, diluted to 0.2 OD/ml in fresh liquid YPD medium, and incubated at 24° with shaking at 250 rpm. The OD_600_ of cultures was measured at 0, 3, 6, 9, 24, and 30 hr. The experiments were performed in triplicate, and data were plotted using Prism 5.03. Strains tested were wild-type (CBS138), *cna1* mutant (YC98), *cnb1* mutant (YC193), and *crz1* mutant (YC182). (C) Calcineurin is required for survival at 40°. Cells were grown overnight in YPD at 24°, 5-fold serially diluted, spotted onto YPD. The plate was incubated at 40° for 48 hr, then transferred to 24° for 48 hr incubation. (D) Calcineurin mutant cells exhibit a shrunken cell morphology at 40°. Cells were grown overnight in YPD medium at 24°, washed twice with dH_2_O, diluted to 0.5 OD_600_/ml in fresh liquid YPD medium, and incubated at 24° or 40° with shaking at 250 rpm for 4 hr. Strains tested were wild-type (CBS138), *cna1* mutant (YC98), *cnb1* mutant (YC193), and *crz1* mutant (YC182). The images were taken at 100×. Scale bar = 5 µm. (E) An osmotic stabilizer rescued temperature-sensitive phenotypes of calcineurin mutants from *C. glabrata*, but not from *C. neoformans*. Cells were grown overnight in YPD at 24°, 5-fold serially diluted, spotted onto YPD medium containing 1 M sorbitol, and incubated at the temperatures indicated for 48 hr. *C. neoformans* strains tested were wild-type (H99) and *cna1* mutant (KK1).

Fungal pathogens must adapt to elevated temperatures and other environmental stresses to successfully infect their mammalian hosts. How these environmental cues are sensed is not clear. In the human fungal pathogen *C. neoformans*, calcineurin mutants are unable to grow at 37° and as a result are avirulent in an animal model of cryptococcal meningitis (Odom *et al.* 1997). In the protozoan parasite *L. major*, a calcineurin mutant (*cnb*) loses viability at 34° and thus fails to survive in mice (Naderer *et al.* 2011). To date, a requirement of calcineurin for high-temperature growth has not been reported in ascomycetes, including species such as *S. cerevisiae* (Cyert *et al.* 1991), *C. albicans* (Bader *et al.* 2006), *A. fumigatus* (Steinbach *et al.* 2006), and *M. oryzae* (Choi *et al.* 2009). Here, we demonstrate that high-temperature growth in *C. glabrata* is controlled by calcineurin and Crz1. *C. glabrata* calcineurin mutants (*cna1* and *cnb1*) exhibited a temperature-sensitive (ts) phenotype at 40°, whereas *crz1* mutants exhibited intermediate growth between the wild-type strain and calcineurin mutants (Figure 1A), suggesting the calcineurin pathway governs growth at elevated temperature. The growth of the wild-type strain in medium containing the calcineurin inhibitors FK506 or cyclosporin A (CsA) at 40° phenocopies the ts growth of calcineurin mutants in the absence of calcineurin inhibitors (Figure 1A). We observed that *cna1* and *cnb1* mutants, and the wild-type strain in the presence of FK506 or CsA, exhibited slight growth defects at 37° but not at 24° (Figure 1A). The growth defects of calcineurin mutants were not simply due to a general reduced rate of growth because wild-type strain and calcineurin pathway mutants had similar growth kinetics at 24° (Figure 1B). Calcineurin mutants were not viable after 48 hr growth at 40° and could not be rescued after transfer to 24° for 48 hr (Figure 1C). When cells were growing in log phase at 40°, *cna1* and *cnb1* mutants exhibited a significantly shrunken cell morphology [65.6 ± 15.3% and 75.0 ± 9.4% of the cellular population, respectively, compared with the wild-type (1.7 ± 1.5%), *P* ≤ 0.002; Figure 1D]. The shrunken cell morphology was also observed in wild-type cells at 40° in the presence of FK506 or cyclosporin A (Figure S1). The *crz1* mutants showed an intermediate proportion of shrunken cells (29.3 ± 9.4%) compared with the wild-type and calcineurin mutants (Figure 1D). Interestingly, the growth defects of the calcineurin mutants at 40° were rescued by the presence of an osmotic stabilizer (1 M sorbitol; Figure 1E, left panel), which is in contrast to *C. neoformans* calcineurin mutant for which the osmotic stabilizer did not rescue the growth of the mutant at 37° (Figure 1E, right panel). Our data suggest a novel role for the calcineurin pathway in controlling thermal stress response of *C. glabrata* and a convergent role of calcineurin in promoting high-temperature growth of *C. glabrata* and *C. neoformans*.

**Figure 2:**
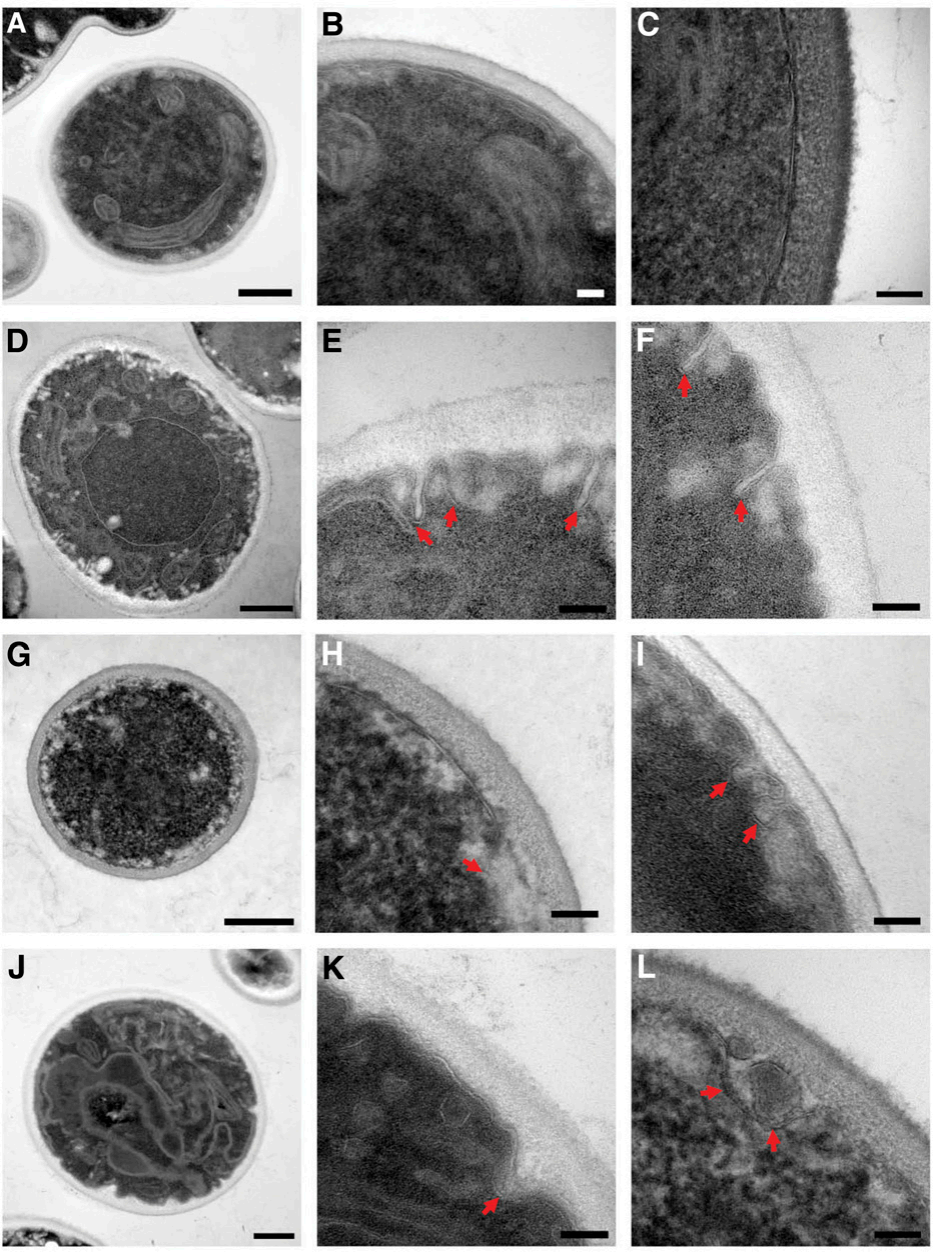
TEM images of *C. glabrata* grown at 37°. Calcineurin mutants (*cna1* and *cnb1*) and *crz1* mutants display aberrant cell membrane structure (red arrowheads) compared with wild-type. (A–C) wild-type (CBS138). (D–F) *cna1* mutant (YC98). (G–I) *cnb1* mutant (YC193). (J–L) *crz1* mutant (YC182). The *cna1* and *cnb1* mutants display the most aberrant phenotypes at 37° and could not be recovered from 40° for TEM analysis. Scale bar = 500 nm (A, D, G, J) and 100 nm (B, C, E, F, H, I, K, L). Column one comprises low-magnification images displaying the overall cell structure. Column two displays a second image, at higher magnification, representative of the cell membrane structure of the cell in column one. Column three displays a high-magnification view of the cell membrane of one additional cell.

The intracellular architecture of calcineurin mutants upon thermal stress in *C. neoformans* and *L. major* remains elusive. Here, we examined cell structures of *C. glabrata* wild-type and calcineurin pathway mutants upon growth at 24°, 37°, and 40° via transmission electron microscopy. *C. glabrata* calcineurin and *crz1* mutants exhibit abnormal cell structures compared with the wild-type at 37° or above (Figure 2 and Figure S2), but not at 24° (data not shown). Calcineurin and *crz1* mutants exhibit irregular plasma membrane structures (red arrowheads in Figure 2, E, H, and K) at 37°, whereas the wild-type strain shows an intact and regular plasma membrane structure (Figure 2B). A similar disruption of the plasma membrane has not been reported for *S. cerevisiae* calcineurin mutants. Because actin forms patches associated with the plasma membrane in *S. cerevisiae* (Doyle and Botstein 1996; Waddle *et al.* 1996), we examined whether *C. glabrata* calcineurin mutants might exhibit defective cortical actin patch structures. However, we found no defects in forming cortical actin patches based on phalloidin staining of calcineurin mutant cells at 37° (Figure S3). Calcineurin and *crz1* mutants show fewer budding cells at 37° compared with the wild-type (data not shown), suggesting a reduced growth rate or cell-cycle arrest of calcineurin pathway mutants. When grown at 40° for 24 hr, calcineurin mutants could not be recovered due to cell death, whereas *crz1* mutants exhibit misshapened organelles (Figure S2C) and irregular plasma membrane structures (Figure S2D) compared with the wild-type. Altogether, our data suggest that the calcineurin pathway is required for maintaining intact plasma membrane structure in *C. glabrata*.

To determine whether calcineurin was required for thermotolerance in all *C. glabrata* strains, we tested the growth of 19 clinical *C. glabrata* isolates at elevated temperatures in the presence of FK506 or CsA. We found that 16% (3/19) of *C. glabrata* clinical isolates exhibit temperature sensitivity in the presence of a calcineurin inhibitor (Figure S4), suggesting that the requirement of calcineurin at elevated temperatures has been evolutionarily diverged in distinct isolates of the species. One of the 3 clinical isolates that exhibited temperature sensitivity in the presence of FK506 or CsA was the type strain and genome-sequenced reference strain CBS138, used as the parental wild-type strain in this study. It is possible that a specific mutation occurred in the genome of the *C. glabrata* ancestor that resulted in reduced fitness, such as a temperature-sensitive response to calcineurin inhibitors secreted by the bacterium *Streptomyces tsukubaensis* in the soil (Goto *et al.* 1987). Because most *C. glabrata* strains did not exhibit the ts phenotype in response to calcineurin inhibitors, it suggests that additional modifier mutations might be present that suppress the ts phenotype. Further studies that isolate high-temperature–resistant mutants from *C. glabrata* CBS138 in the presence of a calcineurin inhibitor and screening suppressors or sequencing the whole genome will shed light on the roles of *C. glabrata* calcineurin in thermotolerance.

### Calcineurin contributes to tissue colonization in a murine urinary tract infection model

**Figure 3:**
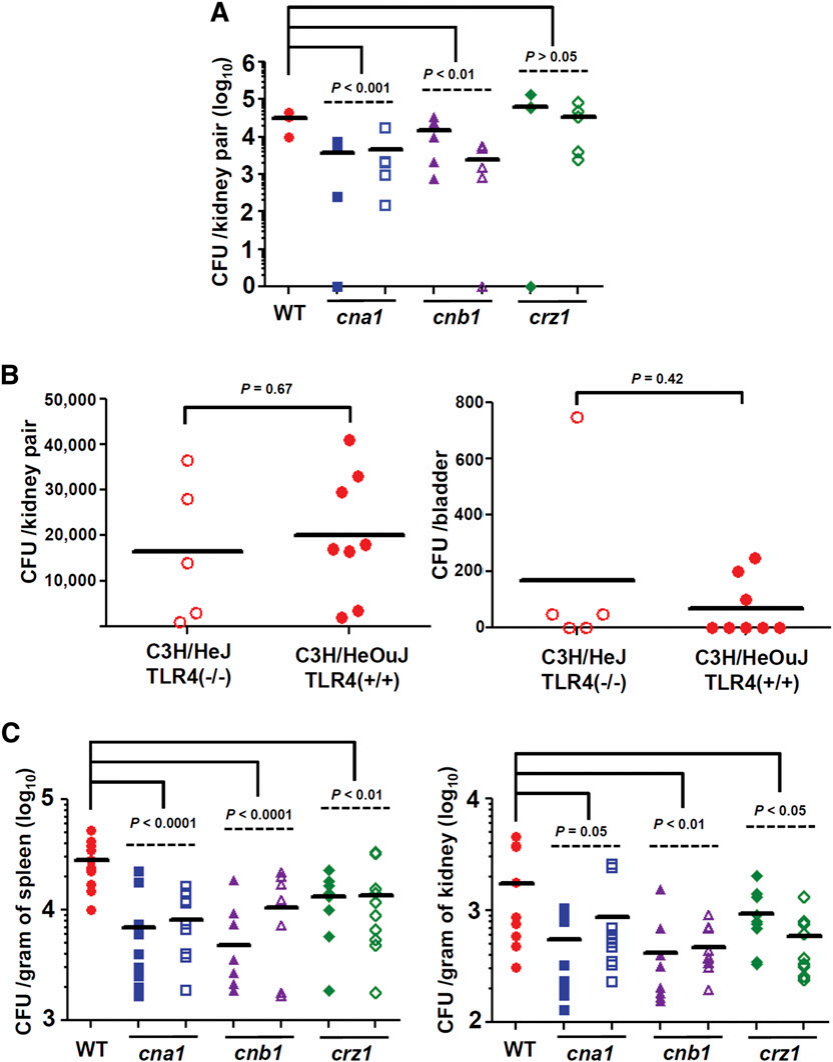
*C. glabrata* calcineurin plays critical roles in tissue colonization of murine urinary tract and systemic infection models. (A) The fungal burden in the kidneys was determined at day 7 after challenge with 3 × 10^7^ cells (in 30 μl) via urinary tract infection. Five female C3H/HeJ mice per strain (except four mice with one *crz1* mutant due to a death following anesthesia with pentobarbital) were used. The *P* value between wild-type (WT) and mutants is shown. Strains tested were wild-type (CBS138); *cna1* mutants (YC67 and YC98); *cnb1* mutants (YC191 and YC193); and *crz1* mutants (YC267 and YC182). (B) Mouse TLR4 is not required for protection from *C. glabrata* urinary tract infection. The fungal burden in the kidneys and bladders was determined at day 7 after challenging C3H/HeJ (group of five animals) or C3H/HeOuJ (group of eight animals) mice with 3 × 10^7^ *C. glabrata* wild-type (CBS138) cells via urinary tract infections. The *P* value between C3H/HeJ and C3H/HeOuJ mice is shown. (C) The fungal burden in the spleen and kidneys was determined at day 7 after challenge with 4 × 10^7^ cells (in 200 µl) via lateral tail vein injection. Ten male CD1 mice per strain were used. The *P* value between WT and mutants is shown.

*Candida* species account for 10–15% of all nosocomial urinary tract infections related to indwelling catheters (Lundstrom and Sobel 2001). *C. albicans* (52%), *C. glabrata* (16%), *C. tropicalis* (8%), and *C. parapsilosis* (4%) were the major *Candida* species found in the urine of 861 patients with funguria (Kauffman *et al.* 2000). Recently, Silva *et al.* (2010) reported that *C. glabrata* strains exhibited a better ability to colonize silicone surfaces in the presence of urine than *C. tropicalis* or *C. parapsilosis*, which may explain why *C. glabrata* is an emerging pathogen in hospitalized and catheterized patients. *C. glabrata* epithelial adhesin triple knockout mutant (*epa1,6,7*) has been demonstrated to have a decreased ability to colonize the bladder in the murine urinary tract infection model (Cormack *et al.* 1999). However, other factors that might contribute to urinary tract infections due to *C. glabrata* are unclear. We demonstrated that calcineurin contributed to murine urinary tract infections. *C. glabrata cna1* and *cnb1* mutants exhibited a 7.5-fold (*P* < 0.001) and 3.7-fold (*P* < 0.01) reduced fungal burden in the kidneys, respectively, whereas *crz1* mutants exhibited no difference compared with the wild-type strain (Figure 3A). Nevertheless, we were unable to determine the fungal burden in bladders when we used the C3H/HeJ inbred mouse strain, which serves as a vesico-ureteric reflux (VUR) model (Murawski *et al.* 2010) and is the only mouse model that has worked for this purpose in our laboratory. VUR is defined by the retrograde passage of pathogen from the bladder into the ureters and pelvis of the kidneys. The percentage of VUR with the *C. glabrata* infection in C3H/HeJ mice was 91.2% (31/34), indicating that most *C. glabrata* cells migrated from the bladder to the kidneys; therefore, the fungal burden in the bladders remained very low due to the nature of this mouse model (Figure S5A). The mouse strains CD1 and CBA/J have been used to determine the fungal burden of *C. glabrata* in bladders and kidneys, but the model was unsuccessful due to poor colonization of the bladder and kidneys with percentages of VUR of 34% and 50%, respectively (data not shown).

### Mouse TLR4 is not required for protection from *C. glabrata* urinary tract infection

C3H/HeJ mice have a Toll-like receptor 4 (TLR4) mutation. TLR4 is a type of pattern recognition receptor (PRR) that recognizes lipopolysaccharides from Gram-negative bacteria and initiates innate immunity. Recently, Leal *et al.* (2010) showed that killing of *A. fumigatus* was reduced in a TLR4(−/−) murine keratitis model, suggesting that TLR4 is required for *A. fumigatus* infection. In *C. albicans*, the role of mammalian TLR4 for *Candida* recognition or infection varies in different studies (Gil and Gozalbo 2006; Netea *et al.* 2006) and appears to be dependent upon the *C. albicans* isolates tested (Netea *et al.* 2010). However, the role of TLR4 against *C. glabrata* infection is unclear. Our data demonstrated that *C. glabrata* can establish infection in kidneys of TLR4(−/−) C3H/HeJ mice following urinary tract inoculation but not in other TLR4(+/+) CD1 or CBA/J mice. To determine whether TLR4 has role in *C. glabrata* infection, we used TLR4(+/+) C3H/HeOuJ mice, which have a similar genetic background as C3H/HeJ mice. Our experiments showed that the *C. glabrata* wild-type strain CBS138 exhibited a similar fungal burden in the kidneys and bladders of TLR4(+/+) C3H/HeOuJ and TLR4(−/−) C3H/HeJ mice in a urinary tract infection model (Figure 3B), suggesting that TLR4 does not modulate *C. glabrata* infection in the urinary tract infection model. Therefore, the differential responses to *C. glabrata* urinary tract infection in CD1, CBA/J, and C3H/HeJ mice might be attributable to specific mouse strains but not to the TLR4 mutation.

### Calcineurin and Crz1 influence tissue colonization in a murine systemic infection model

The requirement of *C. albicans* calcineurin for mammalian virulence depends on the specific niche (Bader *et al.* 2006). For example, calcineurin controls virulence in murine systemic infections, but not in vaginal or pulmonary infection models (Bader *et al.* 2006). In addition, fungal calcineurin uses different pathogenesis mechanisms to cause infections in the host depending on the infection location (Chen *et al.* 2010a). We tested whether *C. glabrata* calcineurin is required for murine systemic infections. Unlike *C. albicans* and *C. dubliniensis*, *C. glabrata* preferentially colonizes the spleen rather than kidney tissues. In splenic tissues, both *cna1* and *cnb1* mutants exhibited a 3.7-fold reduced fungal burden (*P* < 0.0001), whereas *crz1* mutants had a 2.1-fold reduced fungal burden (*P* < 0.01) (Figure 3C) compared with the wild-type strain. The results of the calcineurin mutants in spleen tissues were similar to those reported by Miyazaki *et al.* (2010). However, we found that *crz1* mutants exhibited reduced fungal burden in the spleen, while Miyazaki and colleagues reported no difference. The differential role of *crz1* mutants in colonizing the spleen between the two groups might be due to differences in mouse strains, *C. glabrata* strains, or experimental procedures. In kidney tissues, *cna1* and *cnb1* mutants exhibited 2.5-fold (*P* = 0.05) and 3.9-fold (*P* < 0.01) reduced fungal burdens compared with the wild-type strain, whereas *crz1* mutants showed a 2.3-fold reduced fungal burden (*P* < 0.05) compared with the wild-type strain. Our results of fungal burden in the kidneys were consistent with those reported by Miyazaki *et al.* (2010). Taken together, *C. glabrata* calcineurin and Crz1 contribute to virulence in a murine systemic infection model.

### Calcineurin pathway mutants are unable to establish murine ocular infection

**Figure 4:**
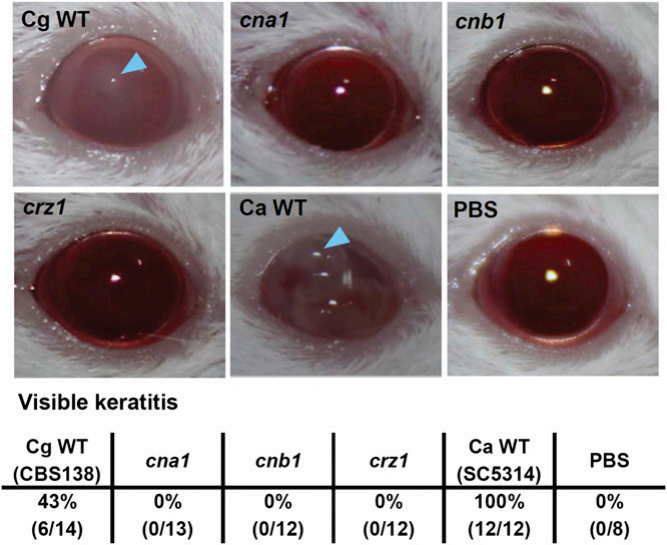
Calcineurin pathway mutants are unable to establish murine ocular infections. Representative clinical photographs of corneas of immunocompetent ICR mice 8 days after challenge with 10^6^ cells of *C. glabrata* wild-type (Cg WT; CBS138), *cna1* (YC98), *cnb1* (YC193), *crz1* (YC182), *C. albicans* wild-type (Ca WT; SC5314), or mock inoculation with PBS. All strains were grown overnight at 25° and diluted with PBS prior to topical application. Fungal keratitis, indicated by blue arrowheads, was seen only in animals infected with *C. glabrata* CBS138 (43%) or *C. albicans* SC5314 (100%). No infection with the calcineurin pathway mutants resulted in visible keratitis in mice.

*C. glabrata* is an uncommon yeast pathogen in ocular infections, but it has recently been reported with increasing frequency in post-keratoplasty patients, often resulting in ocular morbidity, including loss of vision (Al-Assiri *et al.* 2006; Caldwell *et al.* 2009; Grueb *et al.* 2006; Muzaliha *et al.* 2010). However, the mechanisms of ocular infection due to *C. glabrata* have not been studied. We used a murine ocular infection model to investigate the roles of the *C. glabrata* calcineurin pathway in ocular pathogenesis. The *C. glabrata* wild-type strain CBS138 established an ocular infection in 43% (6/14) of the immunocompetent CD1 mice, whereas the *C. albicans* wild-type strain SC5314 established infection in 100% (12/12) of the mice (Figure 4). Ocular infections due to *C. albicans* were significantly more severe than those caused by *C. glabrata* at all time points (*P* < 0.05; Figure S5B), representing the first comparison between *C. albicans* and *C. glabrata* in a murine ocular infection model. *C. glabrata cna1*, *cnb1*, and *crz1* mutants were unable to establish infection, suggesting that the calcineurin pathway controls ocular pathogenesis.

*C. glabrata* wild-type strain CBS138 can establish murine ocular infection in immunocompetent mice, which is in contrast to the inability of *C. dubliniensis* to cause ocular infection in an immunocompetent mouse model (Chen *et al.* 2011). Because *C. albicans* successfully infected the cornea of immunocompetent mice (100%), we conclude that the relative ability of the three *Candida* species to cause murine ocular infection is *C. albicans* > *C. glabrata* > *C. dubliniensis*.

### Calcineurin is essential for ER stress tolerance and cell wall integrity in *C. glabrata*

A straightforward explanation for the attenuated virulence of calcineurin mutants is their growth defects (plasma membrane disruption and slower growth) at body temperature (∼37°). However, it is possible that calcineurin is involved in other mechanisms necessary for the establishment and maintenance of infections. The maintenance of the ER stress response is required for successful infection by fungal pathogens. For example, Hac1, a transcription factor that controls the ER stress response and unfolded protein response (UPR), is required in *A. fumigatus* for virulence in a murine invasive aspergillosis model (Richie *et al.* 2009). The UPR is activated to induce molecular chaperones involved in protein folding upon the accumulation of unfolded or misfolded proteins in the lumen of the ER. Recently, Cheon *et al.* (2011) demonstrated that the *C. neoformans* UPR pathway (*IRE1* and *HXL1*) is essential for ER stress tolerance, thermotolerance, and virulence in a murine intranasal infection model. Because *C. glabrata* calcineurin mutants exhibit similar stress responses to those of *C. neoformans* UPR pathway mutants, it is possible that calcineurin might function together with the UPR pathway to orchestrate multiple phenotypes in *C. glabrata*. Thus far, it is unclear whether the ER stress response is required for virulence of *C. glabrata*.

**Figure 5:**
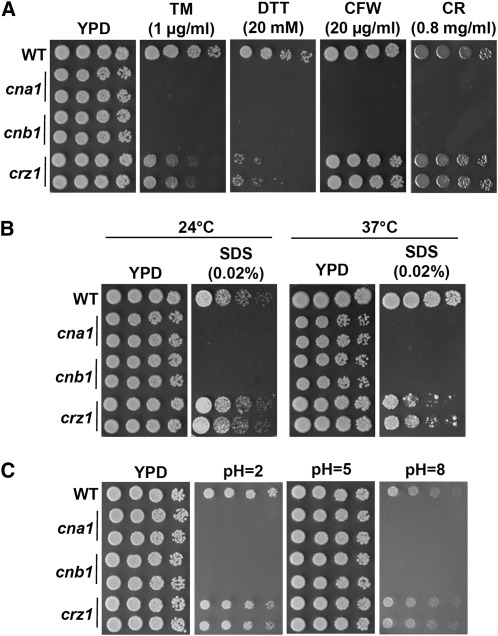
Calcineurin controls ER stress tolerance, cell wall integrity, and pH homeostasis in *C. glabrata*. (A) Calcineurin mutants are hypersensitive to ER stress chemicals and cell wall integrity-damaging agents. Cells were grown overnight in YPD at 24°, 5-fold serially diluted, and spotted onto YPD medium containing tunicamycin (TM), dithiothreitol (DTT), calcofluor white (CFW), or Congo red (CR), and incubated at 24° for 48 hr. (B) Calcineurin and *crz1* mutants are hypersensitive to the cell membrane integrity-damaging agent sodium dodecyl sulfate (SDS). Cells were grown overnight in YPD at 24°, 5-fold serially diluted, and spotted onto YPD medium containing SDS and incubated at indicated temperatures for 48 hr. (C) Calcineurin is required for growth at acidic and alkaline environments. Cells were grown overnight in YPD at 24°, 5-fold serially diluted, and spotted onto YPD medium containing 150 mM HEPES buffered at pH 2 or 5. For pH 8 medium, YPD was buffered with 150 mM pH 9 HEPES (85 ml of YPD plus 15 ml of 1M HEPES at pH 9) due to slightly acidic YPD medium. The pHs of solid media were confirmed with pH indicator strips.

A link between ER stress response and calcineurin is known in *S. cerevisiae* (Bonilla *et al.* 2002), in the plant fungal pathogen *Ustilago hordei* that causes covered smut of barley (Cervantes-Chavez *et al.* 2011), and in the protozoan parasite *L. major*, which causes leishmaniasis in humans (Naderer *et al.* 2011). For example, *U. hordei cna1* and *cnb1* mutants were hypersensitive to the chemical ER stressors tunicamycin (TM; blocks the synthesis of N-linked glycoproteins in the ER) and dithiothreitol (DTT; disrupts the formation of disulfide bonds and leads to retention of proteins in the ER) (Cervantes-Chavez *et al.* 2011). Because Crz1, a downstream target of calcineurin, has not been identified in *U. hordei* or other basidiomycetes, it will be interesting to investigate whether this ER stress response is mediated by Crz1. We demonstrated that the ER stress response is associated with calcineurin and Crz1 function in *C. glabrata*. The *cna1* and *cnb1* mutants exhibited hypersensitive growth on media containing TM or DTT, whereas *crz1* mutants showed intermediate phenotypes between the wild-type strain and calcineurin mutants (Figure 5A).

There is limited information linking ER stress and cell wall integrity in fungi. In *S. cerevisiae*, ER stress has been demonstrated to activate cell wall integrity (CWI) signaling via mitogen-activated protein kinase Slt2/Mpk1 (Krysan 2009; Scrimale *et al.* 2009). In *Candida*, *Cryptococcus*, and *Aspergillus* species, calcineurin is required for cell wall integrity (Chen *et al.* 2010a). Here, we showed that calcineurin but not *crz1* mutants are hypersensitive to agents that damage the cell wall or membrane, including calcofluor white, Congo red, and SDS at 24° (Figure 5, A and B). In accord with TEM images (Figure 2), calcineurin and *crz1* mutants are hypersensitive to the membrane-damaging agent SDS at 37° (Figure 5B). The cell wall integrity of *Candida* species has been demonstrated to be required for virulence in murine systemic infection models (Chen *et al.* 2010b). Thus, the requirement of calcineurin in cell wall/membrane integrity may affect the pathogenicity of *C. glabrata*.

### Calcineurin controls pH homeostasis in *C. glabrata*

How fungal pathogens sense environmental pH changes is central to virulence. In *C. albicans*, calcineurin appears to be required for pH homeostasis (Kullas *et al.* 2007). However, the requirement of calcineurin for pH homeostasis had not been studied in *C. glabrata*. We found that *cna1* and *cnb1* mutants were hypersensitive to both acidic (pH 2) and alkaline (pH 8) conditions (Figure 5C), whereas *crz1* mutants exhibited sensitivity similar to the wild-type, suggesting pH homeostasis is governed solely by calcineurin and not Crz1 in *C. glabrata*.

The *C. glabrata* GPI-anchored aspartyl protease Yps1 has been demonstrated to be required for pH homeostasis (Bairwa and Kaur 2011) and virulence in a murine systemic infection model (Kaur *et al.* 2007). Miyazaki *et al.* (2011b) showed that transcription of *CgYPS1* was regulated by calcineurin in *C. glabrata*. On the basis of previous studies and ours, we suggest that calcineurin may control pH homeostasis and virulence via Yps1 in *C. glabrata*. The pH of mouse urine is about 5.3, and the pH of blood is nearly 7.2. Because calcineurin is essential for growth at pH 2 and pH 8 but not pH 5 (Figure 5), we suggest that the requirement of calcineurin in pH homeostasis may play a limited role in causing infections.

### Optimal growth in serum is controlled by calcineurin but not Crz1 in *C. glabrata*

**Figure 6:**
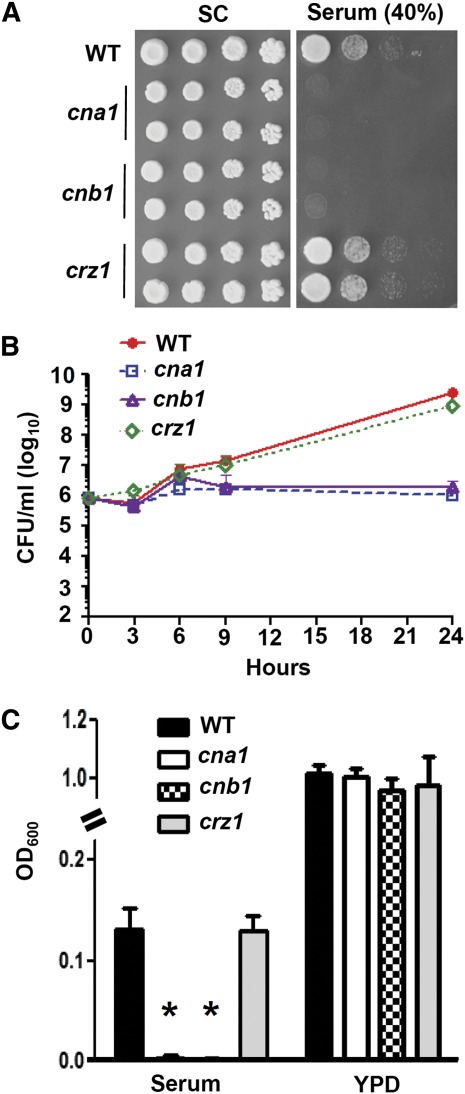
Calcineurin, but not Crz1, is required for optimal growth in serum. (A) Cells were grown overnight in YPD at 24°, 5-fold serially diluted, spotted onto synthetic complete (SC) medium ± 40% fetal bovine serum, and incubated at 24° for 4 days. (B) Time-killing curve of wild-type (CBS138), *cna1* (YC98), *cnb1* (YC193), and *crz1* (YC182) strains grown in 100% serum. Cells surviving at 24° with shaking at 250 rpm after 0, 3, 6, 9, and 24 hr were serially diluted onto YPD medium, and CFUs were counted after 48 hr incubation at 24°. The data are represented as means ± SD from triplicate experiments. (C) Calcineurin mutants cannot proliferate as well as the wild-type strain or *crz1* mutant in serum. Two microliters of 1 OD_600_/ml cells was added to 100 μl of 100% serum or YPD medium in 96-well polystyrene plates, and the OD_600_ was measured after stationary incubation at 24° for 24 hr. The data are represented as means ± SD from triplicate experiments. Asterisk represents *P* < 0.001 compared with the wild-type.

The survival and proliferation of fungal pathogens in host serum are essential for a successful bloodstream infection. The growth defects of calcineurin mutants in serum have been linked to attenuated virulence of *C. albicans* and *A. fumigatus* (Blankenship and Heitman 2005; Steinbach *et al.* 2007). For example, *C. albicans* calcineurin is required for survival in calcium stress, and *A. fumigatus* calcineurin is required for survival in the low-phosphate stress of serum (Chen *et al.* 2010a). Because *C. glabrata* calcineurin mutants exhibited attenuated virulence in a murine systemic infection, we investigated whether serum inhibits the growth of *C. glabrata* calcineurin mutants. Calcineurin, but not *crz1*, mutants are hypersensitive to serum-containing medium (Figure 6A). We also performed a time-kill assay to determine whether serum is lethal to calcineurin mutants. *C. glabrata* calcineurin mutants were not killed in serum, but their growth was arrested, whereas the growth of the *crz1* mutant in serum is similar to that of the wild-type strain (Figure 6B), suggesting the growth arrest in serum was linked to calcineurin but not to Crz1. The addition of the calcium chelators bis(o-aminophenoxy)ethane tetraacetic acid (BAPTA) or ethylene glycol tetraacetic acid (EGTA) to serum did not rescue the growth of calcineurin mutants (data not shown), indicating *C. glabrata* employs calcineurin in a mechanism distinct from *C. albicans* to proliferate in serum. In a liquid growth assay in a polystyrene plate, calcineurin mutants exhibited reduced growth in serum compared with the wild-type strain or *crz1* mutants, whereas in control YPD medium alone there was no difference in growth between the calcineurin mutants and the wild-type strain (Figure 6C). Our data also support the roles of calcineurin in a murine systemic infection model because attenuated virulence of the calcineurin mutants may be in part due to growth arrest in serum.

### Antifungal drug susceptibility is controlled by calcineurin and Crz1 in *C. glabrata*

**Figure 7:**
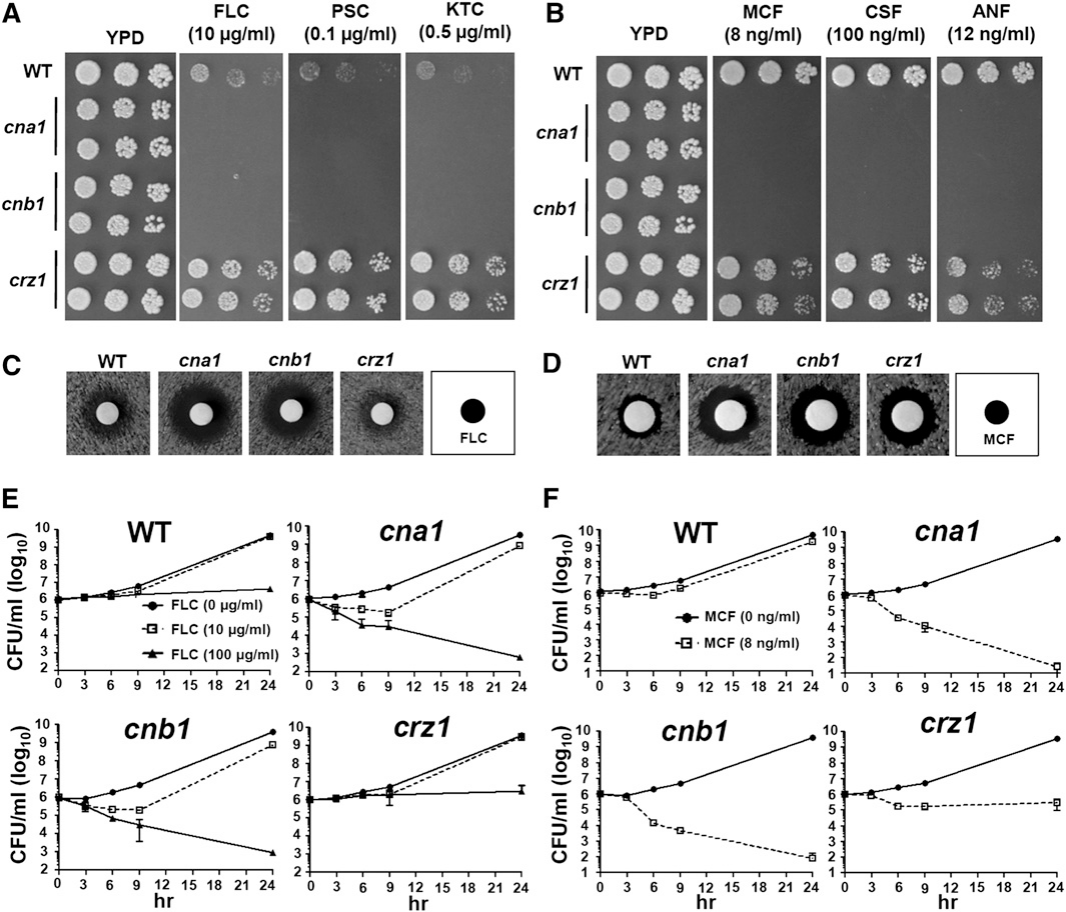
Calcineurin is required for azole and echinocandin tolerance in *C. glabrata*. (A, B) Calcineurin mutants are hypersensitive to azoles and echinocandins. Cells were grown overnight in YPD at 24°, 5-fold serially diluted, spotted onto YPD medium containing fluconazole (FLC), posaconazole (PSC), ketoconazole (KTC), micafungin (MCF), caspofungin (CSF), or anidulafungin (ANF), and incubated at 24° for 48 hr. (C, D) Disk diffusion assays were used to determine fluconazole and micafungin susceptibility of wild-type and mutant strains. Cells were grown overnight at 24°, and 0.1 OD_600_ (in 100 μl) was spread on the surface of YPD medium. A disk containing 20 μg of fluconazole (C) or 50 ng of micafungin (D) was placed on the surface of the medium. The plates were incubated at 24° for 24 hr. (E, F) Time-killing curve of wild-type and *cna1* (YC98), *cnb1* (YC193), and *crz1* (YC182) mutants in YPD medium ± fluconazole (E) or micafungin (F). The experiments were performed at 24°. The data are represented as means ± SD from triplicate experiments.

*C. glabrata* is notorious for its antifungal drug tolerance, which contributes to difficulty in treating infections associated with this species. Our studies demonstrated that calcineurin contributes to virulence in murine urinary tract, systemic, and ocular infections due to *C. glabrata*. Thus, *C. glabrata* calcineurin is a drug target worthy of investigation. Calcineurin mutants (*cna1* and *cnb1*) are hypersensitive to the azoles fluconazole, posaconazole, and ketoconazole (Figure 7A), and to the echinocandins micafungin, caspofungin, and anidulafungin (Figure 7B). In disk diffusion assays, calcineurin mutants exhibit larger and clearer zones of inhibition in the presence of fluconazole or micafungin than the wild-type strain (Figure 7, C and D). To confirm the fungicidal effects of the drugs on calcineurin mutants, we performed time-kill assays. Fluconazole exhibited fungicidal activity at 100 µg/ml, but not at 10 µg/ml, against calcineurin mutants over 24 hr of incubation (Figure 7E). At a concentration of 8 ng/ml, micafungin showed fungicidal activity against calcineurin mutants but not the wild-type strain (Figure 7F). Taken together, our data suggest that combination therapy with a calcineurin inhibitor and fluconazole or micafungin may improve the treatment outcome of *C. glabrata* infections.

Interestingly, *crz1* mutants showed differential responses to azoles and echinocandins. Unlike the intermediate hypersensitivity phenotypes of *crz1/crz1* mutants in *C. albicans* and *C. dubliniensis* (Chen *et al.* 2011), *C. glabrata crz1* mutants exhibit hyperresistance to azoles (Figure 7A), suggesting that Crz1 might repress genes involved in azole tolerance in *C. glabrata*. In response to echinocandins, *crz1* mutants showed an intermediate phenotype between the wild-type and the calcineurin mutants (Figure 7B), suggesting that echinocandin tolerance is mediated by a calcineurin-dependent Crz1 pathway.

### Genome-wide analysis of calcineurin- and Crz1-dependent targets in *C. glabrata*

**Figure 8:**
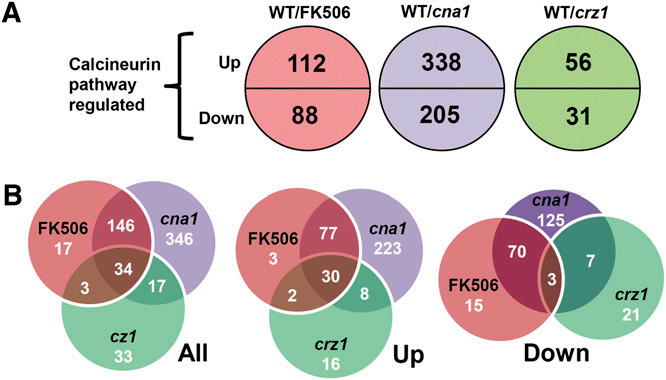
Genome-wide analysis of calcineurin- and Crz1-dependent targets in *C. glabrata*. Strains were grown overnight at 24° and washed twice with dH_2_O. Cells were diluted to 0.2 OD/ml in YPD and incubated at 24° for 3 hr. For the wild-type strain, cells in log-phase were diluted to 0.2 OD/ml (10 ml) in YPD in the presence or absence of FK506 (1 μg/ml). *cna1* (YC98) and *crz1* (YC182) mutants were diluted to 0.2 OD/ml (10 ml) in YPD. Following 3 hr incubation at 37°/250 rpm, cells were collected for total RNA extraction, microarray experiments, and subsequent analysis. (A) Calcineurin pathway up- and downregulated genes in a *C. glabrata* wild-type strain treated with FK506, *cna1*, and *crz1* mutants. (B) The overlaps between regulated genes for an FK506-treated wild-type strain, *cna1*, and *crz1* mutants are depicted as Venn diagrams.

To better understand the multiple phenotypes of calcineurin pathway mutants and to identify calcineurin pathway–regulated genes in *C. glabrata*, we compared the genome-wide transcriptome profile of a wild-type strain with an FK506-treated wild-type strain, a *cna1* mutant, and a *crz1* mutant (Figure 8A). We expected that the FK506-treated wild-type strain would have a similar expression profile to that of the *cna1* mutant. The genes regulated by FK506 largely overlapped (90%, 180/200) with differentially expressed genes in a *cna1* mutant (Figure 8B). By looking at calcineurin-upregulated and calcineurin-downregulated genes, the FK506 group overlapped 96% (107/112) and 83% (73/88), respectively, with the *cna1* mutant (Figure 8B). Interestingly, 38% (33/87) of Crz1-dependent genes are Cna1-independent (Figure 8B), indicating that proteins other than calcineurin might regulate Crz1 and result in differential expression profiles compared with the calcineurin-dependent Crz1 pathway. The genes regulated by FK506 exhibited 29% (32/112, upregulated) and 3% (3/88, downregulated) overlap, respectively, with differential expressed genes in a *crz1* mutant (Figure 8B), suggesting a strong correlation between FK506-treated wild-type and *cna1* mutant, but not with the *crz1* mutant. In addition, FK506 treatment of wild-type cells resulted in differential expression of 20 genes (Table S2) distinct from those in the *cna1* mutant, including genes encoding plasma membrane ATP-binding cassette (ABC) multidrug transporters (*PDR5*, *PDR15*, and *YOR1*), the bile acid transporter *YBT1*, and the transcription factor *INO2*, possibly due to FK506 inhibition of FKBP12 or other FKBPs or to direct action on multidrug resistance (MDR) pumps.

**Figure 9:**
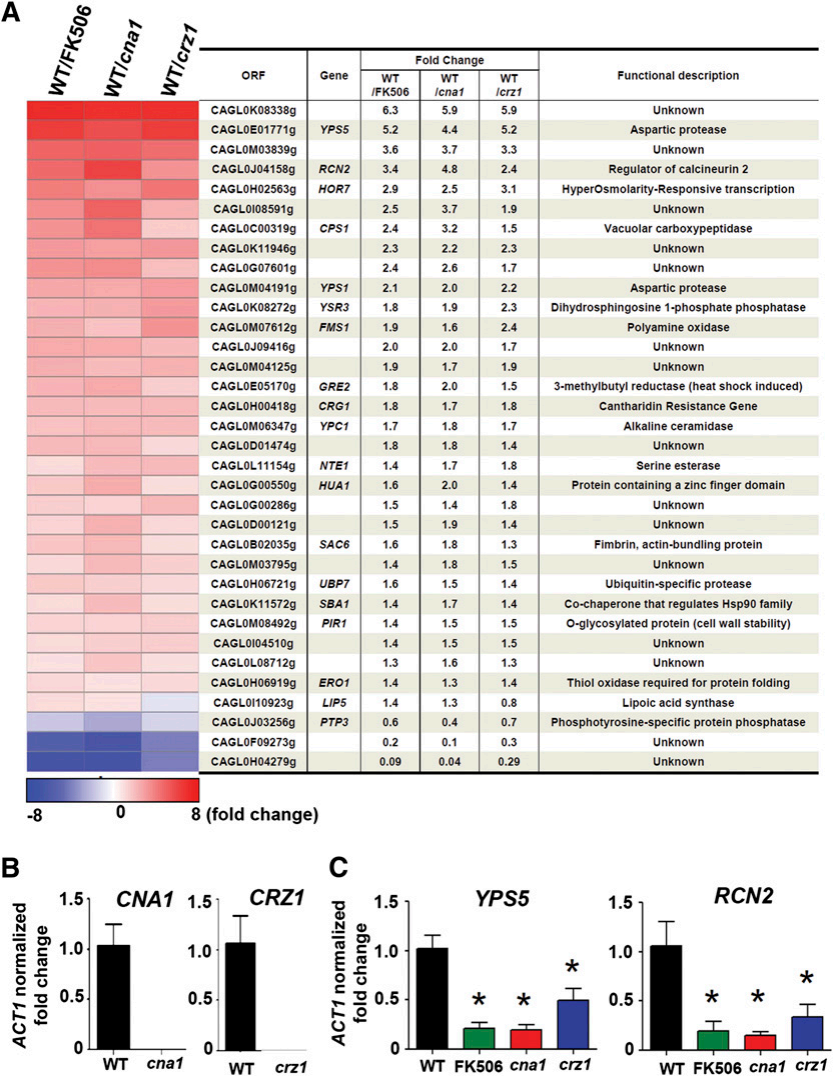
Genes regulated by the calcineurin pathway in *C. glabrata*. (A) List of 34 genes regulated by calcineurin and Crz1. There are 30 genes that were upregulated by calcineurin and Crz1, 3 genes downregulated by calcineurin and Crz1, and 1 gene (*LIP5*) upregulated by calcineurin but downregulated by Crz1. The criteria used to classify genes as upregulated or downregulated in the microarray experiments is a cutoff Q value < 0.001. (B) The transcription of *CNA1* and *CRZ1* was abolished in the *cna1* (YC98) and *crz1* (YC182) mutants, respectively. Real-time RT-PCR was used to measure the expression level of each gene. (C) Real-time RT-PCR was used to independently assess the expression levels detected in microarray analyses for *YPS5* and *RCN2* in wild-type, FK506-treated wild-type, *cna1* mutant (YC98), and *crz1* mutant (YC182). Statistically significant differences (*P* < 0.05 based on ANOVA, Bonferroni’s multiple comparison tests) compared with the wild-type are indicated by the asterisks.

Because the requirement of calcineurin in *C. glabrata* is different from *C. albicans* in terms of thermal stress tolerance and optimal serum growth, it is of interest to explore the calcineurin and Crz1 targets in *C. glabrata*. We identified 34 genes that are regulated by both calcineurin and Crz1 (Figure 9A), some of which encode proteins involved in cell wall biosynthesis (*YPS5*, *YPS1*, *YSR3*, *YPC1*, *PIR1*, *HOR7*, and *NTE1*), heat shock protein responses (*SBA1* and *GRE2*), and calcineurin regulation (*RCN2*). Among 34 genes, 30 genes are upregulated and 3 genes are downregulated by the calcineurin pathway, whereas 1 gene (*LIP5*) is upregulated in the FK506-treated wild-type and *cna1* mutant and is downregulated in the *crz1* mutant (Figure 9A). Although the transcription levels of another calcineurin regulator (*RCN1*) and calcineurin binding protein 1 (*CBP1*) have been demonstrated to be upregulated by calcineurin and Crz1 in *S. cerevisiae* and by Crz1 in *M. oryzae* (Kim *et al.* 2010; Yoshimoto *et al.* 2002), it was not regulated by *C. glabrata* calcineurin and Crz1. Our transcript profiling data suggest that the heat shock response genes (*SBA1* and *GRE2*) regulated by calcineurin and Crz1 may be involved in controlling thermal stress tolerance in *C. glabrata*. Consistent with a recent study that *CgYPS1* was regulated by Cnb1 and Crz1 in *C. glabrata* (Miyazaki *et al.* 2011b), our microarray data support this finding and provide a comprehensive view of the transcriptome regulated by the calcineurin pathway. Real-time RT-PCR analysis confirmed that the transcription of *CNA1* and *CRZ1* was abolished in the *cna1* and *crz1* mutants, respectively (Figure 9B). The transcription of two control genes (*YPS5* and *RCN2*) determined by real-time RT-PCR was well correlated with the microarray data (Figure 9C).

### Rcn2 is a feedback inhibitor of calcineurin signaling

**Figure 10:**
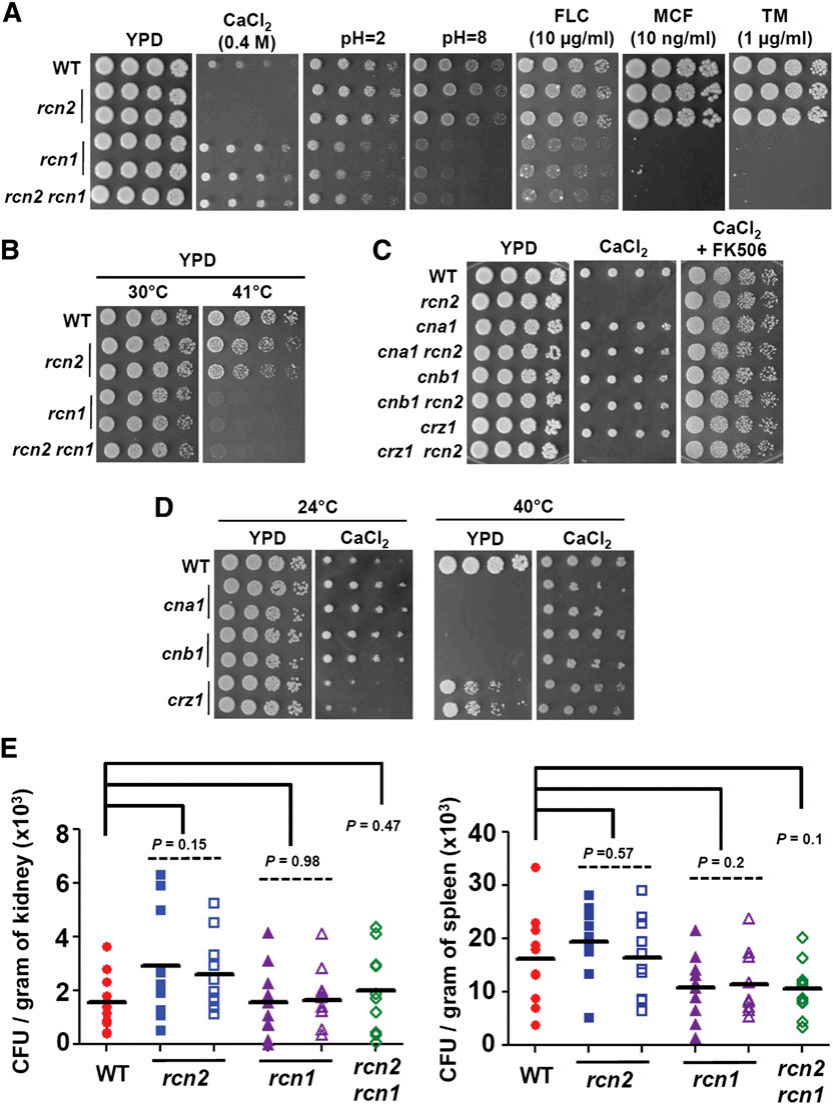
Divergent roles of the calcineurin signaling cascade in stress responses. (A) Rcn2 and Rcn1 have divergent roles in stress responses. Strain preparations were carried out as described above. The plates were incubated at 30° for 48 hr. Strains tested were wild-type (CBS138), *rcn2* mutants (YC525 and YC531), *rcn1* mutants (YC553 and YC556), and the *rcn2 rcn1* double mutant (YC594). (B) Rcn1 contributes to thermotolerance. The plates were incubated at temperatures indicated for 24 hr. (C) Rcn2 acts as a negative regulator of calcineurin signaling in response to calcium stress. Strains spotted onto YPD medium ± CaCl_2_ (0.4 M) and/or FK506 (1 μg/ml) were incubated at 30° for 48 hr. (D) Calcineurin mutants exhibit modest resistance to calcium stress and are rescued by calcium at elevated temperatures. Cells were grown overnight in YPD at 24°, 5-fold serially diluted, spotted onto YPD medium in the absence or presence of 0.4 M CaCl_2_, and incubated at the indicated temperatures for 48 hr. (E) The fungal burden in the spleens and kidneys was determined on day 7 after challenge with 4 × 10^7^ *C. glabrata* wild-type or mutant cells (in 200 µl) via lateral tail vein injection. Ten male CD1 mice per strain were used.

**Figure 11:**
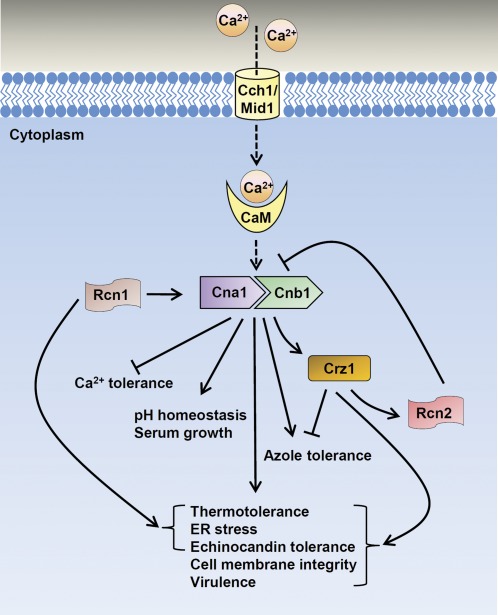
Proposed roles of the calcineurin signaling cascade in stress responses, drug tolerance, and virulence in *C. glabrata*. Calcineurin and Crz1 are required for various stress responses, including thermotolerance, ER stress, echinocandin tolerance, cell membrane integrity, and virulence. Calcineurin also exhibits Crz1-independent functions, including pH homeostasis and optimal growth in serum. Calcineurin (Cna1 or Cnb1) suppresses calcium tolerance, whereas Crz1 inhibits azole tolerance. Regulator of calcineurin 1 (Rcn1) is an endogenous positive regulator of calcineurin that participates in the control of multiple cellular processes, including thermotolerance, ER stress, and echinocandin tolerance. Rcn2 acts as a feedback inhibitor of calcineurin function in calcium tolerance. Solid arrows represent promoting functions, and bar-headed arrows represent inhibitory functions within the calcineurin signaling cascade. The dotted arrow represents function as seen in *S. cerevisiae*, but undetermined in *C. glabrata*.

Regulator of calcineurin 2 (*RCN2/YOR220w*) was first identified a decade ago in *S. cerevisiae* with a genetic screen for endogenous inhibitors of calcineurin (Kingsbury and Cunningham 2000). The transcription of *ScRCN2* is activated by calcineurin and Crz1 (Mehta *et al.* 2009). In *C. albicans*, microarray data showed that the transcription of *CaRCN2* (*IPF12084/ORF19.6554*, function uncharacterized) was activated in a calcineurin- and Crz1-dependent manner (Karababa *et al.* 2006). Miyazaki *et al.* (2011a) recently showed that transcription of *CgRCN2* was regulated by *C. glabrata* Cnb1 and Crz1. According to our microarray analysis and real-time RT-PCR, we independently demonstrated that the transcription of *CgRCN2* was strongly activated by Cna1 and Crz1 in *C. glabrata*. To test whether Rcn2 is a downstream target of calcineurin and Crz1, we constructed *rcn2* mutants and found that calcineurin-controlled cellular functions, such as thermotolerance, drug tolerance, ER stress, and pH homeostasis, were not affected by deletion of *RCN2* (Figure 10, A and B), indicating that Rcn2 is not a functional target of calcineurin in *C. glabrata*. Instead, we found that *rcn2* mutants were hypersensitive to Ca^2+^ (Figure 10, A and C), in contrast to the modestly increased Ca^2+^ tolerance of calcineurin mutants (Figure 10D, left panel). In *S. cerevisiae*, cells lacking calcineurin grow better in the presence of high concentrations of Ca^2+^ compared with wild-type cells (Cunningham and Fink 1994; Withee *et al.* 1998). This result suggests that inhibition of Ca^2+^ tolerance by calcineurin is conserved in *S. cerevisiae* and *C. glabrata*. In contrast, *C. albicans* and *C. dubliniensis* calcineurin mutants are hypersensitive to Ca^2+^ (Chen *et al.* 2011; Sanglard *et al.* 2003), suggesting a divergent role of calcineurin in Ca^2+^ tolerance between *C. albicans*/*C. dubliniensis* and *C. glabrata/S. cerevisiae*. The modestly increased Ca^2+^ tolerance of calcineurin mutants suggests that calcineurin represses Ca^2+^ tolerance in *C. glabrata* (Figure 11). The mechanisms by which genes control calcium tolerance are unclear in *C. glabrata*. Vacuolar Ca^2+^ ATPase (Pmc1) and vacuolar Ca^2+^/H^+^ exchanger (Vcx1) are required for calcium tolerance in *S. cerevisiae* (Cunningham and Fink 1996); therefore, we hypothesize that the homologs of *S. cerevisiae* Pmc1 and Vcx1 also control calcium tolerance in *C. glabrata*, and it is possible that calcineurin inhibits Pmc1 or Vcx1 via dephosphorylation in *C. glabrata*. Interestingly, Ca^2+^ can rescue the temperature-sensitive growth of *C. glabrata* calcineurin mutants at 40° (Figure 10D, right panel), indicating that Ca^2+^ may activate a specific signaling cascade that supports growth at an elevated temperature.

In *S. cerevisiae*, Rcn2 can inhibit calcineurin activity, and truncation mutations that removed the P×I×IT-like motif [P/G]×[I/V]×[I/V/L][E/D/N/H/T] of Rcn2 abolished its inhibition of calcineurin signaling (Mehta *et al.* 2009). We found that *C. glabrata* and *C. albicans* Rcn2 also has a P×I×IT-like motif (Figure S6B), indicating calcineurin inhibition mediated by Rcn2 might be conserved in these three species. Interestingly, the T××P motif required for calcineurin stimulation found in ScRcn1 and CgRcn1 is not found in CaRcn1 (Figure S6A) or in Rcn2 of *S. cerevisiae*, *C. glabrata*, and *C. albicans* (Figure S6). The synteny analysis of Rcn1 and Rcn2 in *S. cerevisiae*, *C. glabrata*, and *C. albicans* suggests that *C. albicans* calcineurin regulators are diverged from *S. cerevisiae* and *C. glabrata* (Figure S7).

The opposite phenotypes between *rcn2* and calcineurin mutants in response to Ca^2+^ led us to hypothesize that Rcn2 might serve as a negative regulator of calcineurin in *C. glabrata*. To test this hypothesis, we generated a set of calcineurin pathway and *rcn2* double mutants. The *cna1 rcn2* and *cnb1 rcn2* (but not *crz1 rcn2*) double mutants exhibited a reversal of the Ca^2+^ hypersensitivity of the *rcn2* mutant, and the addition of FK506 to Ca^2+^-containing media also rescued the growth of *rcn2* mutants (Figure 10C). Taken together, our data suggest that Rcn2 serves as a negative regulator of calcineurin and that calcineurin suppresses calcium tolerance in *C. glabrata* (Figure 10D). Interestingly, Rcn2 is not required for virulence in a murine systemic infection model (Figure 10E).

### Rcn1 controls cellular functions similar to calcineurin in *C. glabrata*

Regulator of calcineurin 1 (Rcn1) was first identified in fungi (Gorlach *et al.* 2000; Kingsbury and Cunningham 2000) and mammals (Fuentes *et al.* 2000; Rothermel *et al.* 2000) on the basis of its ability to interact with and inhibit calcineurin when overexpressed. In *S. cerevisiae*, Rcn1 can also stimulate calcineurin signaling (Kingsbury and Cunningham 2000). Rcn1 was independently identified as Cbp1 in a screen for calcineurin-binding proteins in *C. neoformans* and shown to be required for full virulence in a murine systemic infection model (Gorlach *et al.* 2000). In *C. albicans*, Rcn1 (Ipf13909/Orf19.7770) controls cell membrane integrity and azole tolerance, but not pathogenicity, in murine systemic infection and oropharyngeal candidiasis models (Reedy *et al.* 2009). In contrast to *S. cerevisiae* (Yoshimoto *et al.* 2002), we found that calcineurin and Crz1 do not transcriptionally regulate Rcn1 in *C. glabrata*. It is possible that Rcn1 physically interacts with calcineurin to regulate calcineurin signaling. Thus, Rcn1 and calcineurin may act on shared downstream targets. Miyazaki *et al.* (2011a) recently showed that Rcn1 controls ER stress response and drug tolerance in *C. glabrata*. Our *rcn1* mutants exhibited similar phenotypes and have additional phenotypes, such as hypersensitive growth to altered pH (acidic and alkaline) and elevated temperature (41°) (Figure 10, A and B), and modest resistance to Ca^2+^ (Figure 10A). Unlike *C. neoformans cbp1* mutants, *C. glabrata rcn1* mutants did not exhibit attenuated virulence compared with the wild-type strain in a murine systemic infection model (Figure 10E). Overall, calcineurin mutants exhibit more severe phenotypes than *rcn1* mutants, suggesting that Rcn1 is only partially required for calcineurin function. The *rcn1 rcn2* double mutant had a virulence level similar to the wild-type strain (*P* = 0.1 in spleen; *P* = 0.47 in kidney) in a murine systemic infection model (Figure 10E) and exhibited phenotypes similar to the *rcn1* mutant. This indicates that *rcn1* mutation is epistatic to the *rcn2* mutation in *C. glabrata*. Rcn1 may play a larger role in maintaining calcineurin signaling than the role of Rcn2 in calcineurin inhibition.

In mammalian cells, regulator of calcineurin 1 (DSCR1/RCAN1/calcipressin) is preferentially expressed in the brain of patients with Down’s syndrome and contributes to tumor suppression, suggesting DSCR1 could be explored as a potential therapeutic target (Baek *et al.* 2009). The difference in virulence of *C. neoformans cbp1* and *C. glabrata rcn1* mutants suggests a divergent role of Cbp1/Rcn1 in controlling pathogenicity between *C. neoformans* and *C. glabrata*. Our data also showed that Rcn1 controls azole and echinocandin tolerance (Figure 10A), making drug combination therapy with yet-to-be-developed Rcn1 inhibitors and azoles or echinocandins possible approaches to target emerging *C. glabrata* infections.

## Conclusions

We have demonstrated in this study that calcineurin and Crz1 contribute to the control of thermotolerance, ER stress response, echinocandin tolerance, cell membrane integrity, and pathogenesis in *C. glabrata* (Figure 11). In addition, calcineurin, but not Crz1, contributes to pH homeostasis, serum growth, and azole tolerance (Figure 11). In contrast to *C. albicans*, *C. glabrata* calcineurin modestly inhibits calcium tolerance, suggesting a divergent role of calcineurin in governing calcium tolerance between *C. glabrata* and *C. albicans*. Unlike the role of Crz1 as the positive regulator that controls azole tolerance in *C. albicans*, *C. glabrata* Crz1 acts as a negative regulator of azole tolerance (Figure 11), indicating rewiring of this transcription factor among *Candida* species. The calcineurin regulator Rcn1 positively regulates calcineurin functions, including thermotolerance, ER stress response, and echinocandin tolerance, but not cell membrane integrity and virulence. The other calcineurin regulator, Rcn2, acts as a feedback inhibitor of calcineurin signaling (Figure 11). In the future, determining how calcineurin interacts with other signaling pathways to control core stress responses and how Rcn1 and Rcn2 interact with calcineurin will contribute to our understanding of calcineurin biology and signal transduction.

## Supplementary Material

Supporting Information
