# Correction to: Convergent Evolution of Calcineurin Pathway Roles in Thermotolerance and Virulence in *Candida glabrata*

**DOI:** 10.1093/g3journal/jkaf124

**Published:** 2025-06-09

This is a correction to: Ying-Lien Chen, Jay H Konieczka, Deborah J Springer, Samantha E Bowen, Jing Zhang, Fitz Gerald S Silao, Alice Alma C Bungay, Ursela G Bigol, Marilou G Nicolas, Soman N Abraham, Dawn A Thompson, Aviv Regev, Joseph Heitman, Convergent Evolution of Calcineurin Pathway Roles in Thermotolerance and Virulence in *Candida glabrata*, *G3 Genes|Genomes|Genetics*, Volume 2, Issue 6, 1 June 2012, Pages 675–691, https://doi.org/10.1534/g3.112.002279

As a result of a random sample of paper analysis, the authors would like to note that in the published version of the paper, that several YPD plate images in this paper were duplicated in multiple figure panels. All of the duplicated figure panels are images of spot dilution assays of fungal cells grown on the surface of agar plates at various growth temperatures. These experiments consisted of multiple control and experimental plates grown under the specified conditions. For illustration purposes, the data was divided into several panels within figures, and in some cases the same experimental data was presented in additional figures for ease of comparing assay results. The images that appear duplicated are the appropriate controls or experimental growth tests for each of the experiments shown. Specifically, the images involved are: 1) YPD spotting assays at 40°C in panels Figure 1A and E, and Figure 10D, 2) YPD spotting assays at 37°C in Figure 1A and Figure 5B, 3) YPD control spotting assays at 24°C in Figure 5A and B, and 4) YPD control spotting assays at 24°C in Figure 7A and B. The authors note that in some cases these images were cropped to present only a portion of the spot dilution assays relevant in making the comparison. The authors apologize for any confusion caused by this mode of data presentation. The editors confirm that these considerations do not impact any of the conclusions of the publication.

