# Supplementary material for: Convergent Evolution of Calcineurin Pathway Roles in Thermotolerance and Virulence in Candida glabrata
Source: G3 (Bethesda). 2012 Jun 1;2(6):675–91. doi: 10.1534/g3.112.002279 (PMC3362297; doi:10.1534/g3.112.002279)
Supplement: Supporting Information [file supp_2_6_675__index.html]

Supporting Information 

# Convergent Evolution of Calcineurin Pathway Roles in Thermotolerance and Virulence in *Candida glabrata*

## Supporting Information for Chen *et al*, 2012

**Files in this Data Supplement:**

- Supporting Information - Figures S1-S7 and Tables S1 and S2 (PDF, 7.9 MB)
- Figure S1 - *C. glabrata* wild-type cells exhibit a shrunken cell morphology at 40�C in the presence of FK506 or CsA (PDF, 916 KB)
- Figure S2 - TEM images of *C. glabrata* wild type (CBS138) and crz1 mutant (YC182) cells grown at 40�C (PDF, 641 KB)
- Figure S3 - *C. glabrata* wild type and calcineurin mutant cells exhibit normal cortical actin patch structures. (PDF, 472 KB)
- Figure S4 - Some *C. glabrata* clinical isolates exhibit temperature-sensitive growth when exposed to calcineurin inhibitors (PDF, 691 KB)
- Figure S5 - Virulence of *C. glabrata* calcineurin and *crz1* mutants in murine urinary tract and ocular infection models (PDF, 197 KB)
- Figure S6 - Pairwise alignment of calcineurin regulators from *C. glabrata*, *S. cerevisiae*, and *C. albicans* (PDF, 703 KB)
- Figure S7 - Synteny analysis of Rcn1 and Rcn2 (PDF, 1.1 MB)
- Table S1 - PCR primers (PDF, 919 KB)
- Table S2 - Genes induced by FK506 but not by calcineurin mutation (PDF, 710 KB)
