# Supplementary material for: Convergent Evolution of Calcineurin Pathway Roles in Thermotolerance and Virulence in Candida glabrata
Source: G3 (Bethesda). 2012 Jun 1;2(6):675–91. doi: 10.1534/g3.112.002279 (PMC3362297; doi:10.1534/g3.112.002279)
Supplement: Supporting Information [file supp_2.6.675_002279SI.pdf]

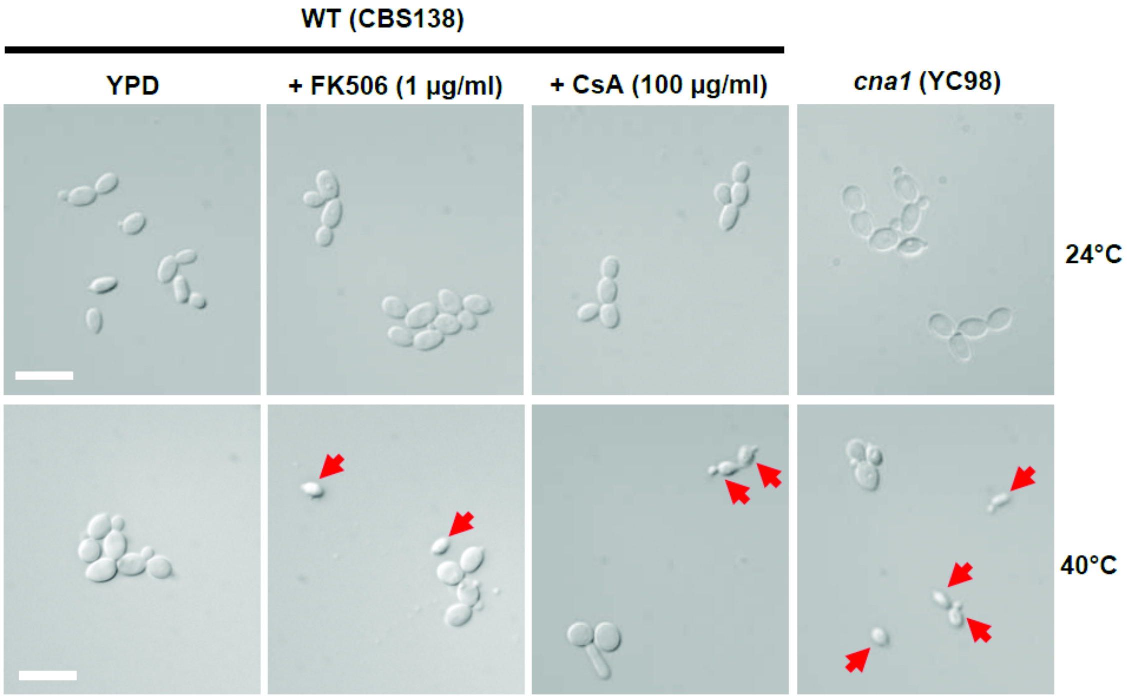

Figure S1 *C. glabrata* wild-type cells exhibit a shrunken cell morphology at 40°C in the presence of FK506 or CsA. Cells were grown overnight in YPD medium at 24°C, washed twice with dH_2O, diluted to 0.5 OD_{600}/ml in fresh liquid YPD medium in the presence or absence of FK506 (1 µg/ml) or CsA (100 µg/ml), and incubated at 24°C or 40°C with shaking at 250 rpm for 4 h. The *cna1* mutant strain was served as the control. The shrunken cells (marked with arrowheads) were only found at high temperature (40°C), but not at 24°C. The percentage of shrunken cells at 40°C in wild-type, FK506-treated wild-type, CsA-treated wild-type, and *cna1* mutant are 3.1 ± 1.4% (mean ± standard deviation; from three independent experiments), 27.1 ± 8.3%, 23.4 ± 9.3%, and 42 ± 11.3%, respectively. The images were taken at 100X. Scale bar = 10 µm.

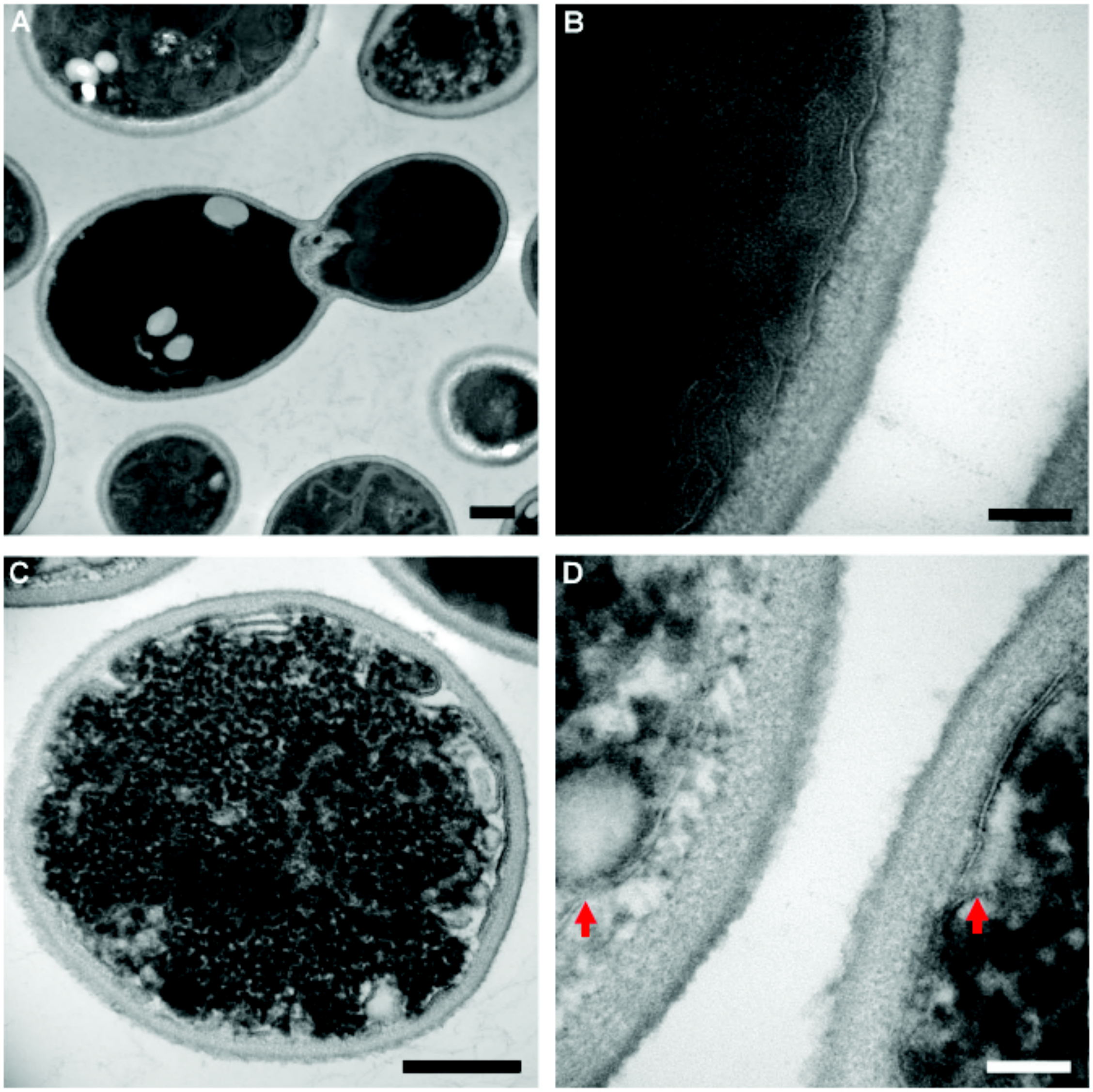

Figure S2 TEM images of *C. glabrata* wild type (CBS138) and *crz1* mutant (YC182) cells grown at 40°C. *C. glabrata cna1* and *cnb1* could not be recovered from growth at 40°C for TEM analysis. Wild-type cells **(A, B)** display normal morphology and budding but *crz1* mutants **(C, D)** display aberrant cell membrane and morphology in comparison to wild-type cells. Scale bar = 500 nm **(A, C)** and 100 nm **(B, D)**. First and second columns represent a global image of the cell and a higher magnification view of the cell membrane, respectively.

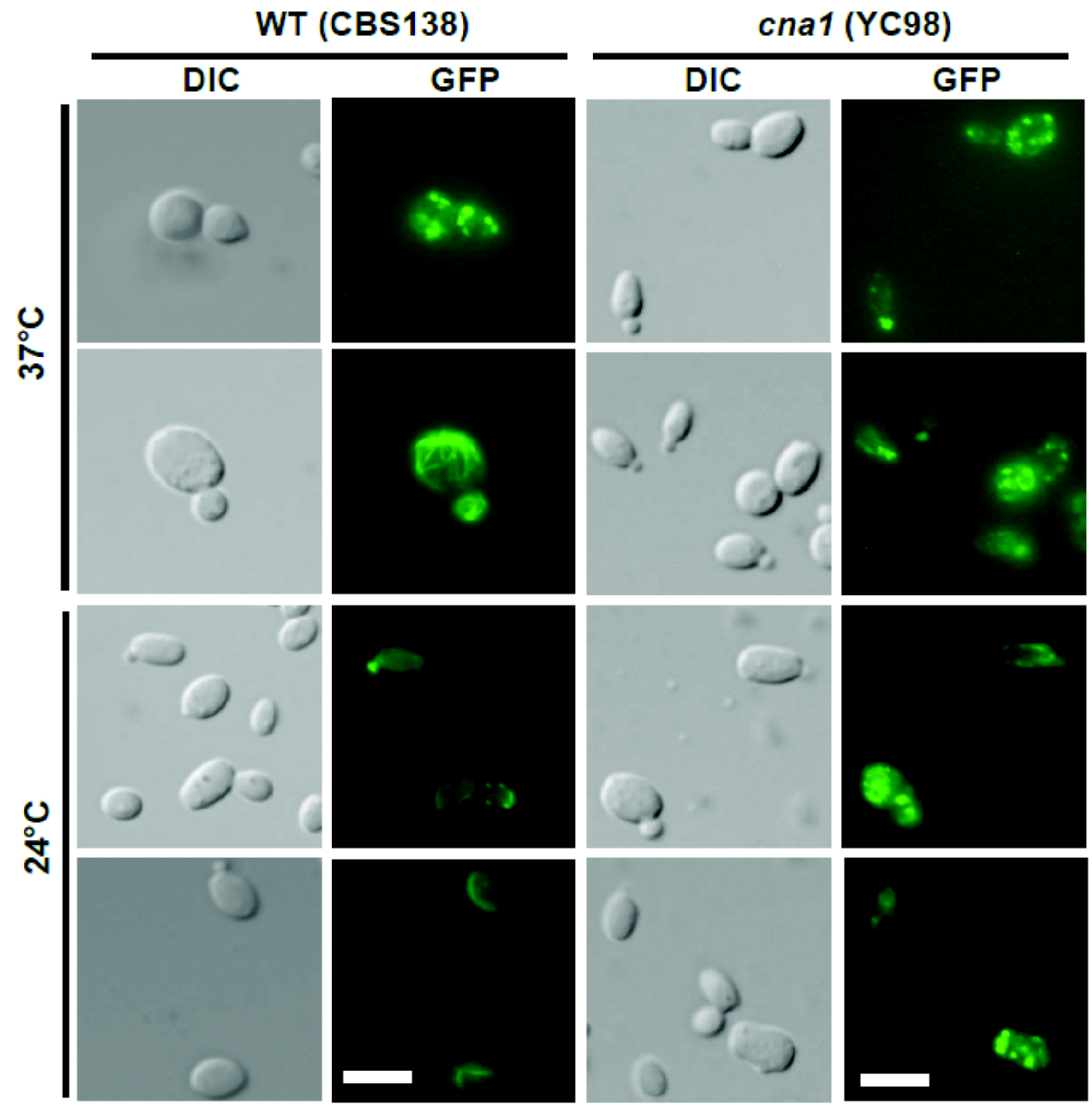

Figure S3 *C. glabrata* wild type and calcineurin mutant cells exhibit normal cortical actin patch structures. The incubation and staining of cells with Alexa fluor® 488 phalloidin are described in material and methods. The images were taken at 100X. Scale bar = 5 µm.

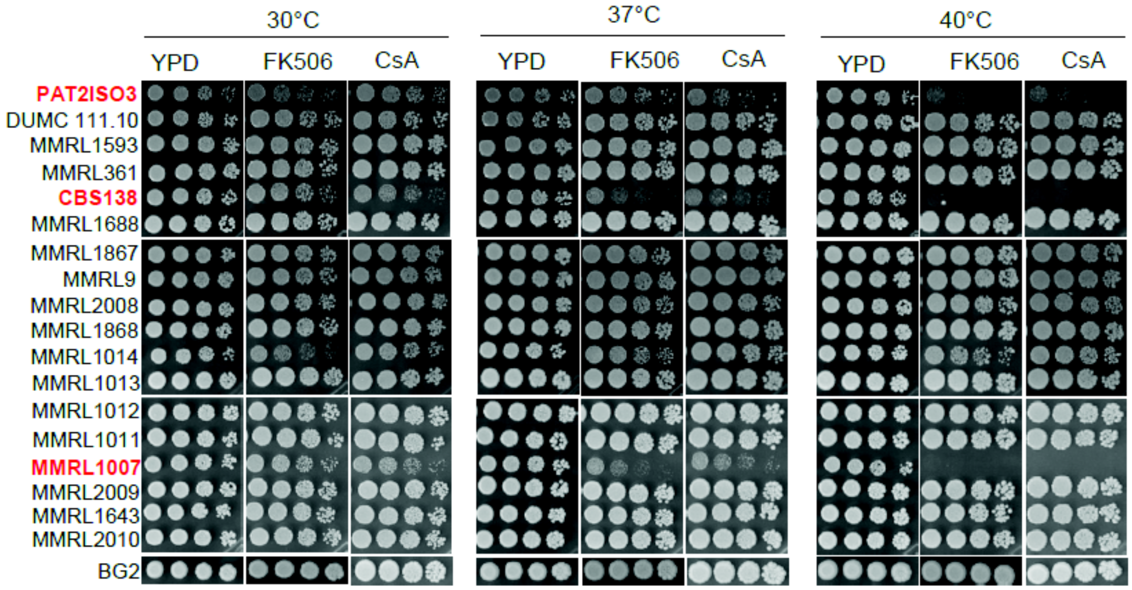

Figure S4 Some *C. glabrata* clinical isolates exhibit temperature-sensitive growth when exposed to calcineurin inhibitors. Cells were grown overnight in YPD at 30°C, 5-fold serially diluted, and spotted onto YPD medium containing FK506 (1 µg/ml) or cyclosporin A (CsA; 100 µg/ml). Cultures were incubated at the indicated temperatures for 48 h. *C. glabrata* strains that are hypersensitive to calcineurin inhibitors at high temperature are indicated in dark red.

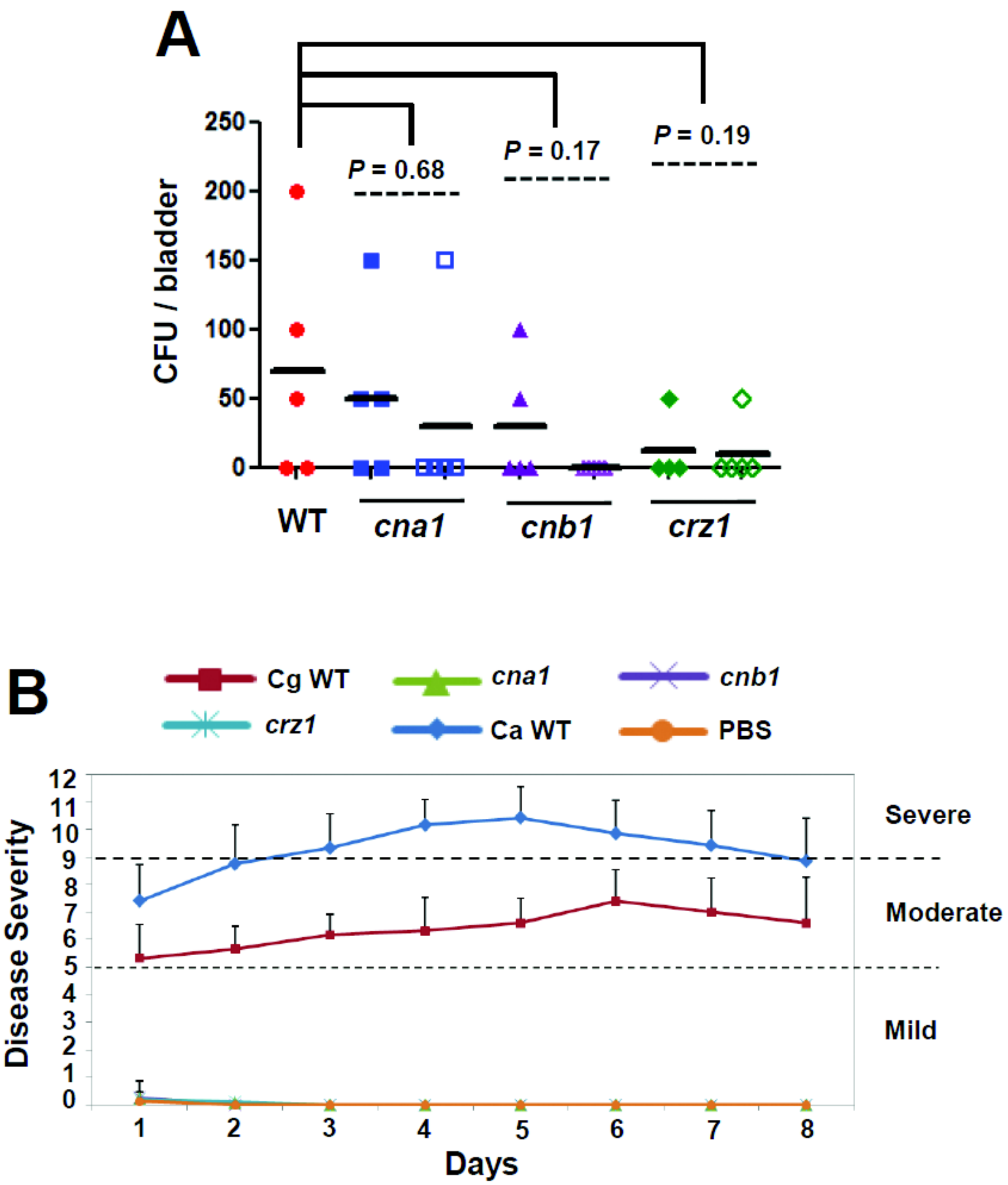

Figure S5 Virulence of *C. glabrata* calcineurin and *crz1* mutants in murine urinary tract and ocular infection models. **(A)** The fungal burden in the bladders was determined at day 7 after challenging mice with 3 x 10^7 cells via urinary tract infection. Five female C3H/HeJ mice were inoculated per strain (except one *crz1* mutant with 4 mice due to the death of one mouse following anesthesia treatment). The *P* value between wild-type (WT) and mutants is shown. **(B)** Disease severity was scored for 8 days. *C. albicans* SC5314 and the PBS mock inoculation served as reference controls. Mice infected with *C. glabrata cna1, cnb1,* or *crz1* mutants, or the PBS control, exhibited normal corneas, and score curves essentially overlapped. Mice infected with wild-type *C. glabrata* CBS138 and *C. albicans* SC5314 strains exhibiting visible signs of keratitis were plotted.

```
   1                                                50
A  CgRcn1  MDSLVTDTIILTSNERDITGQEILPILASWFEDNVLSNIQVLPANPIQLL
   ScRcn1  MGNIITDTIIITSDKCDIVDNDNVERIQVWLSKNILRKFQINENEPLQLI
   CaRcn1  MPRKPTNTLIITNIDDSLLSNP--EPIISLLGD-------Q--PYMIELI

   CgRcn1  LLKNFKRCLIICPDHAVSNAVMQKLKL------------------KVPLP
   ScRcn1  ILKRFKRILLICPSHDISQHVMDASR-------------------ALEME
   CaRcn1  VLMKFKRILLYCQTSKIATKTKTFLLNQWETTTIGGGGGGGGGGGSGGGN

   CgRcn1  DLIFNYSITDSQNNSS--------------VKKDYLKVPE--HQRLFLIS
   ScRcn1  NFNFSYSLQDGQRN----------------LTKQYLKVPE--SEKMFLIS
   CaRcn1  SISISYSLKDNSRILDPEEEDEEDGKDGGGKGGRFLDIPKDLEIKRFLIS

   CgRcn1  PPSSPPPEFD-YSKCEDKPS--FKTHAHQPEKILRQAT------------
   ScRcn1  PPASPPPEFD-FSKCEDAPQRHIQSHIQQDQQQRLEASQLLPNPDKNNN
   CaRcn1  PPASPHSEWDDWDKVEEGPN---ETNIHDYLWEKLKTH-----------

   CgRcn1  -SFVAIESQVGTITVNTCDDYN-NGGPGLEG-VRTALPPKSIFDDIDE-
   ScRcn1  GTFTLLKSKVGAITIDRCPTNDGNGQMQLADHVKTAFPPKSIFDTDDDD
   CaRcn1  ------ENEEKEKDSGISEDDD-----GCES-IKKVELPKIVLNATDN-

   1                                                50
B  CgRcn2  -----------MKTQVLITDVPKNKFD--SKWPDTLEKKLFKEKYP---
   ScRcn2  ------MANQKQMRTQILITDIPSGKFT--SKWPTQLEKTLFKEQFP---
   CaRcn2  MSTSPKLSEITIPKTSLIISNLNKEDFVKPSSSQDSQSTKLISKNLALVD

   CgRcn2  -----------QYRKHLQYFTPLPFLDRVIIILLDDEAATMEIYEYLQKEE
   ScRcn2  -----------NLQSHLQYYTPLPFLNRIIIIFDNEDDTLQVFKFLQ-EL
   CaRcn2  QIKLKVLNFEDDIASHISHWSNLPFLSRVIVIFQNETTAKKIFEFLTKEL

   CgRcn2  PSVVNDKS-IKLYLTESLLSNRF-RSRSMGESSPEDSNAASNGSADSNSA
   ScRcn2  LAKENSGP-MKLFVTESLLNNQHPRSRSTDDAVSLQDNNLALLEDHRN--
   CaRcn2  AKFDDHYSYIKIHLQENLLQKSK-SSDNLHDCQNEKLNVTKSLNNFRNFH

   CgRcn2  IG-DKPILSLDTNPVSTGINVQ-SLAMGSPSLSPDRRTSLESPTLLKFAN
   ScRcn2  ----KPLLSINTDPGVTGVDSS-SLNKGGSSLSPDK-SSLESPTMLKLST
   CaRcn2  NDPKNEFTSEYVEPEPAKFNVYNDLANLGIDLSQYN-SAEQLEELKQSQQ

   CgRcn2  DEKAHLYREPLPTKSDSSK----------------------ESSPGTTE
   ScRcn2  DSKPFSYQEPLPKLSRSSSSTSNLSLNRSSQTSLPSQLENKDKSASGTKC
   CaRcn2  DQNQDQNQKEVKNSRDGNSHSN--------TNSLPVQLG---RRKSTTKT

   CgRcn2  LHKSQ--KLTVSTDAVNKMASNTP---------PPLSPSITVDEFFN
   ScRcn2  LFASKPLGLTIDTSTRSNAASCTENDVNATASNPPKSPSITVNEFFH
   CaRcn2  LFKPE---LKLNTTAKPGLLNEKQKE-----QDFVSSPTITLDETF-
```

Figure S6 Pairwise alignment of calcineurin regulators from *C. glabrata*, *S. cerevisiae*, and *C. albicans*. Multiple sequence alignments are depicted using ClustalW software (http://npsa-pbil.ibcp.fr/cgi-bin/npsa_automat.pl?page=npsa_clustalw.html). The conserved amino acids and TxxP motif are purple- and green-shaded, respectively. The PxIxIT-like motif [P/G]x[I/V]x[I/V/L][E/D/N/H/T] is blue-shaded in panel A (depicting Rcn1 alignments), and underlined in panel B (depicting Rcn2 alignments).

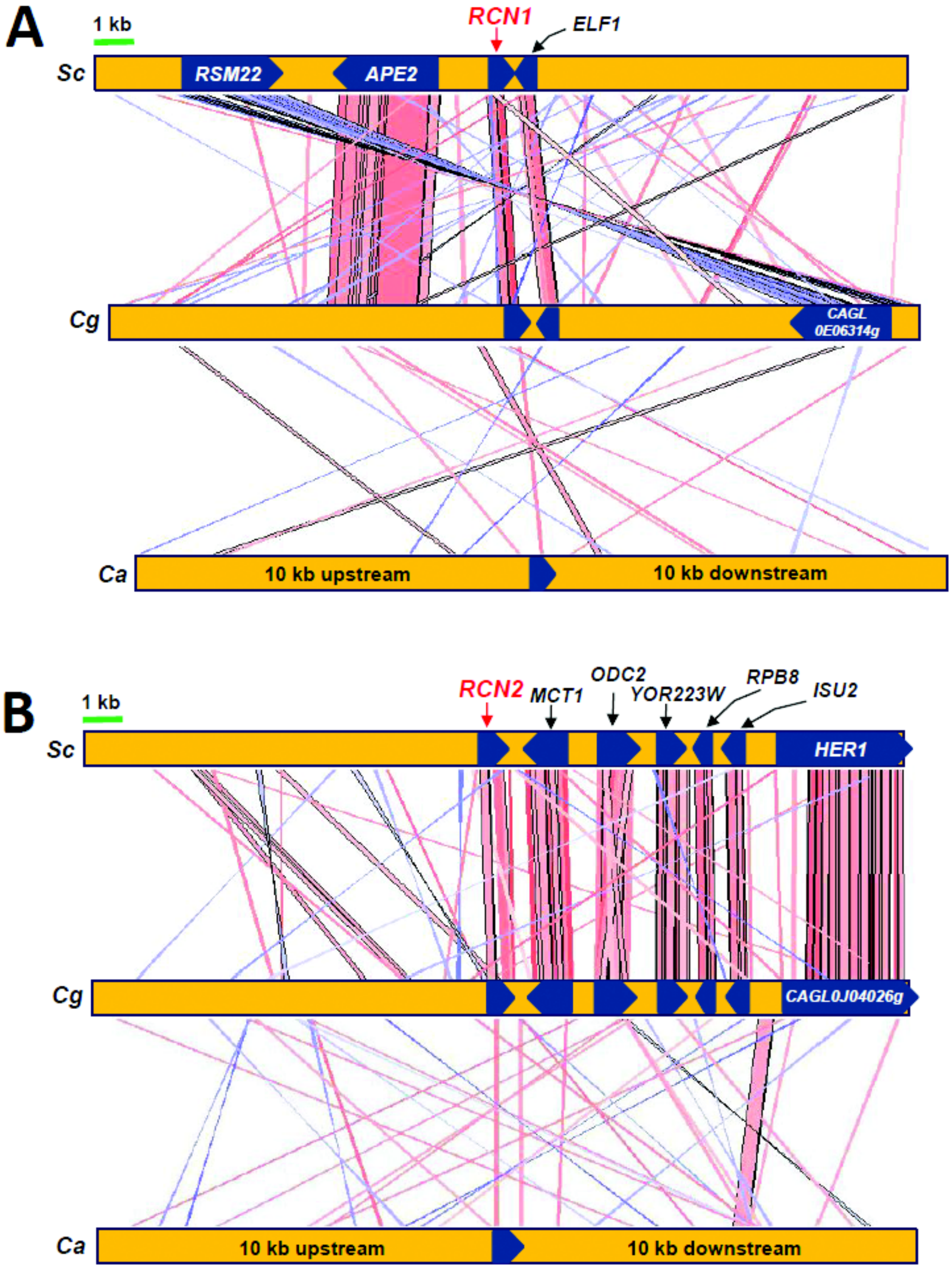

Figure S7 Synteny analysis of Rcn1 and Rcn2. NCBI tBLASTx (Altschul *et al.*, 1997) and Artemis programs (Carver *et al.*, 2008) were used to conduct synteny analysis. Red and blue blocks (lines) represent forward and reverse matches of the DNA sequence (tBLASTx based) among *S. cerevisiae* (*Sc*), *C. glabrata* (*Cg*), and *C. albicans* (*Ca*).

REFERENCES

Altschul, S.F., Madden, T.L., Schaffer, A.A., Zhang, J., Zhang, Z., Miller, W., and Lipman, D.J. (1997) Gapped BLAST and PSI-BLAST: a new generation of protein database search programs. *Nucleic Acids Res* **25**: 3389-3402.

Carver, T., Berriman, M., Tivey, A., Patel, C., Bohme, U., Barrell, B.G., Parkhill, J., and Rajandream, M.A. (2008) Artemis and ACT: viewing, annotating and comparing sequences stored in a relational database. *Bioinformatics* **24**: 2672-2676.

Table S1 PCR primers

Primer	Use	Sequence (5' → 3')
JC17	*SAT1* flipper	GGCCCCCCCTCGAGGAAGTT
JC18	*SAT1* flipper	GCTCTAGAACTAGTGGATCT
JC49	5'NCR of *CNA1*	ACACATTATGCAACATCAA
JC50	5'NCR of *CNA1*	AACTTCCTCGAGGGGGGGCCATTGTCGGTGCCAACAAGAA
JC51	3'NCR of *CNA1*	AGATCCACTAGTTCTAGAGCTTTCATCTCAAGAGCAAAA
JC52	3'NCR of *CNA1*	TTGAACGTTACTGGGGTTGA
JC55	*CNA1* ORF	AAGACAGACGCAGAGCTGAAT
JC56	*CNA1* ORF	GACATTCGTTTTTGAAAGTGA
JC48	Disruption confirmation	ACAATCAAAGGTGGTCCT
JC81	Disruption confirmation	AACTTCCTCGAGGGGGGGCC
JC122	Disruption confirmation	CACACTTTTATTGTTGTCGCC
JC134	5'NCR of *CNB1*	ACACCATAAAAAGTCCCAGCG
JC135	5'NCR of *CNB1*	AACTTCCTCGAGGGGGGGCCTTCCCTTGATTAATACTTCTC
JC136	3'NCR of *CNB1*	AGATCCACTAGTTCTAGAGCGGCAAGAAACTTAACGAGGA
JC137	3'NCR of *CNB1*	TCAACTTCAACAGAGTAGCTC
JC138	*CNB1* overlap	CAAACGTTTTGTTGGATGGC
JC139	*CNB1* overlap	CAACACTTTCGGAGAGAGAT
JC140	*CNB1* ORF	ATGGGAGCTGCACCATCTAAA
JC141	*CNB1* ORF	TACTGAAGGGTCAGGCTCTTT
JC142	5'NCR of *CRZ1*	TAAACCCATGGAGTGTGGAA
JC143	5'NCR of *CRZ1*	AACTTCCTCGAGGGGGGGCCGCTGAATATTGCAAAATCTTG
JC144	3'NCR of *CRZ1*	AGATCCACTAGTTCTAGAGCCACAAACCTCCAGTATTTTT
JC145	3'NCR of *CRZ1*	TCAAGTCTTAGAGATTCACCA
JC146	*CRZ1* overlap	TAATGAAAGCAATGCCAA
JC147	*CRZ1* overlap	GCAACAAATTTCTTGACTGGT
JC148	*CRZ1* ORF	TGGGCGATAACGAAGAGGATA
JC149	*CRZ1* ORF	CTCAAGTTATTTGAAGATGCA
JC441	5'NCR of *RCN2*	CCGTCGGGCTTCTTTAAAT
JC442	5'NCR of *RCN2*	AACTTCCTCGAGGGGGGGCCTTGTGCTGCTATATCTGCGTG
JC443	3'NCR of *RCN2*	AGATCCACTAGTTCTAGAGCGGGGATTGCTTCTATGAAGCT
JC444	3'NCR of *RCN2*	AAATTGCTCACGGAGTCACCT
JC445	*RCN2* overlap	CTCGGGATCATTGCTCAATA
JC446	*RCN2* overlap	AACGGGTCTAGTCGAGGACTT
JC453	*RCN2* ORF	AAAGGATCCATGAAGACACAGGTATTGA
JC454	*RCN2* ORF	AAAGGGCCCCTAGTTAAAGAACTCATCGAC
JC459	5'NCR of *RCN1*	CGAGTGTTTATTATTCGGG
JC460	5'NCR of *RCN1*	AACTTCCTCGAGGGGGGGCCTCCGATAAGAATGTTCAGATG
JC461	3'NCR of *RCN1*	AGATCCACTAGTTCTAGAGCGTACCTATCATGGTTGGTTAA
JC462	3'NCR of *RCN1*	TGTGATCTGCTCGATTTTACC
JC463	*RCN1* overlap	CCATTAACTATAGACGTTGCT
JC464	*RCN1* overlap	AACAGCACATAGAAAGATCAA
JC465	*RCN1* ORF	ACTCACGTCTAATGAAAGGGA
JC466	*RCN1* ORF	ATTTTGGTGGCAATGCCGTT
JC370	qPCR *ACT1* ORF	GTACCACCATGTTCCCAGGT
JC371	qPCR *ACT1* ORF	ACCACCGATCCAGACAGAGT
JC437	qPCR *CNA1* ORF	ACAGAGCGCTGTTCCTGATT
JC438	qPCR *CNA1* ORF	TGGTTGGGAAGTCCTGTTTT
JC439	qPCR *CRZ1* ORF	GGGCGATAACGAAGAGGATA

JC440	qPCR *CRZ1* ORF	TACATCGCCATTCATGCTGT
JC431	qPCR *YPS5* ORF	CAAGATCAGCGATGAACTAACG
JC432	qPCR *YPS5* ORF	TACTTGCGCTGACAAACCAC
JC435	qPCR *RCN2* ORF	CAGTACTTCACGCCGCTTC
JC436	qPCR *RCN2* ORF	CGATAACAGGGACTCCGTCA

Underline indicates sequences complementary to the *SAT1* flipper

Table S2 Genes induced by FK506 but not by calcineurin mutation.

ORF	Gene	Fold change (FK506 / WT)	Functional description
CAGL0M12947g		10.2	Unknown
CAGL0M01760g	*PDR5*	9.5	Plasma membrane ATP-binding cassette multidrug transporter
CAGL0M09713		5.3	Unknown
CAGL0F02717g	*PDR15*	2.8	Plasma membrane ATP-binding cassette multidrug transporter
CAGL0E06688g		2.3	Unknown
CAGL0K09702g		2.2	Unknown
CAGL0C03223g	*SDH2*	1.9	Iron-sulfur protein subunit of succinate dehydrogese
CAGL0M09735g	*MEC3*	1.9	Damage and meiotic pachytene checkpoint protein
CAGL0M01870g		1.8	Unknown
CAGL0G00242g	*YOR1*	1.8	Plasma membrane ATP-binding cassette multidrug transporter
CAGL0C03289g	*YBT1*	1.6	Bile acid transporter
CAGL0K12958g		1.4	Unknown
CAGL0B01947g	*INO2*	1.3	Heteromeric Ino2/Ino4 basic helix-loop-helix transcription activator
CAGL0L09603g	*DSN1*	1.3	Important for chromosome segregation
CAGL0C04433g	*MCD1*	1.3	Required for sister chromatid cohesion in mitosis and meiosis
CAGL0I04180g	*CUP2*	0.8	Copper-binding transcription factor
CAGL0M09889g		0.7	Unknown
CAGL0J08184g	*ALP1*	0.7	Arginine transporter
CAGL0G06358g	*SNC1*	0.7	Vesicle membrane receptor protein
CAGL0A01089g		0.6	Unknown
