# Supplementary material for: Convergent Evolution of Calcineurin Pathway Roles in Thermotolerance and Virulence in Candida glabrata
Source: G3 (Bethesda). 2012 Jun 1;2(6):675–91. doi: 10.1534/g3.112.002279 (PMC3362297; doi:10.1534/g3.112.002279)
Supplement: Supporting Information [file supp_2.6.675_FigureS1.pdf]

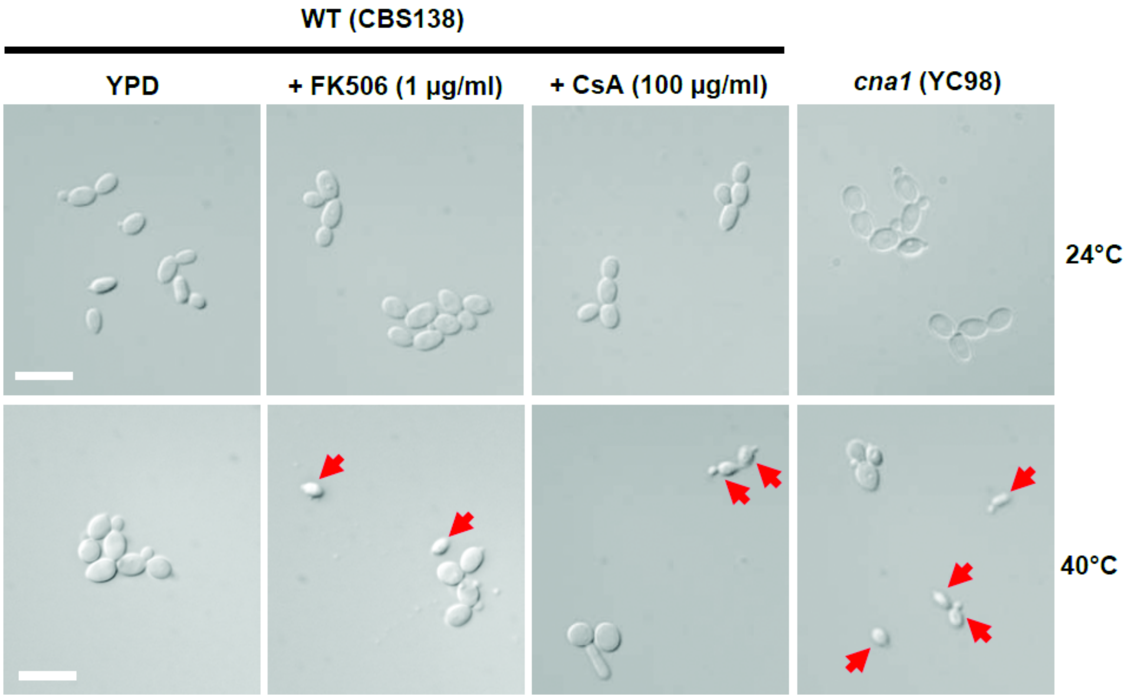

Figure S1 *C. glabrata* wild-type cells exhibit a shrunken cell morphology at 40°C in the presence of FK506 or CsA. Cells were grown overnight in YPD medium at 24°C, washed twice with dH_2O, diluted to 0.5 OD_{600}/ml in fresh liquid YPD medium in the presence or absence of FK506 (1 µg/ml) or CsA (100 µg/ml), and incubated at 24°C or 40°C with shaking at 250 rpm for 4 h. The *cna1* mutant strain was served as the control. The shrunken cells (marked with arrowheads) were only found at high temperature (40°C), but not at 24°C. The percentage of shrunken cells at 40°C in wild-type, FK506-treated wild-type, CsA-treated wild-type, and *cna1* mutant are 3.1 ± 1.4% (mean ± standard deviation; from three independent experiments), 27.1 ± 8.3%, 23.4 ± 9.3%, and 42 ± 11.3%, respectively. The images were taken at 100X. Scale bar = 10 µm.
