# Supplementary material for: Convergent Evolution of Calcineurin Pathway Roles in Thermotolerance and Virulence in Candida glabrata
Source: G3 (Bethesda). 2012 Jun 1;2(6):675–91. doi: 10.1534/g3.112.002279 (PMC3362297; doi:10.1534/g3.112.002279)
Supplement: Supporting Information [file supp_2.6.675_FigureS2.pdf]

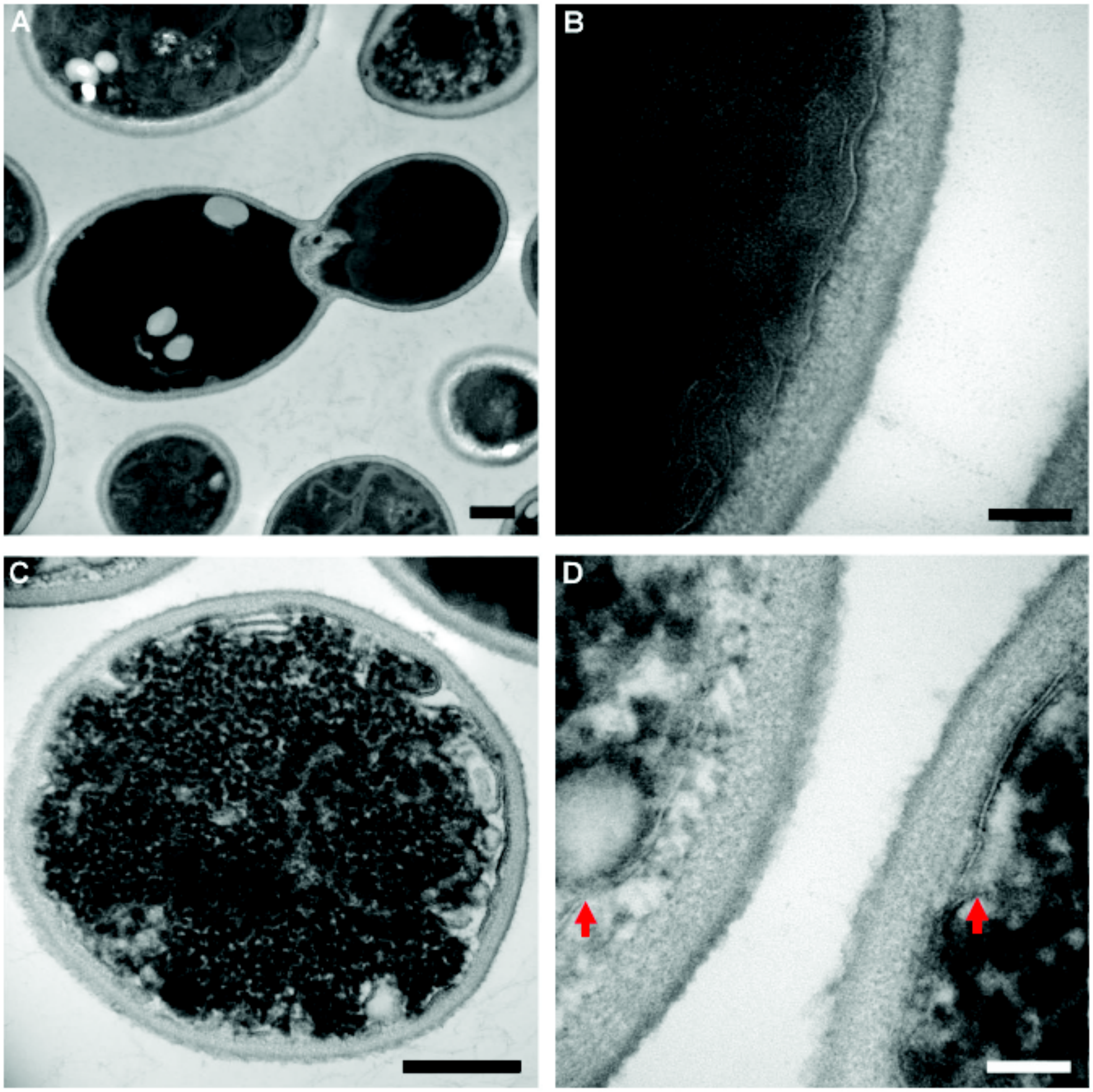

Figure S2 TEM images of *C. glabrata* wild type (CBS138) and *crz1* mutant (YC182) cells grown at 40°C. *C. glabrata cna1* and *cnb1* could not be recovered from growth at 40°C for TEM analysis. Wild-type cells **(A, B)** display normal morphology and budding but *crz1* mutants **(C, D)** display aberrant cell membrane and morphology in comparison to wild-type cells. Scale bar = 500 nm **(A, C)** and 100 nm **(B, D)**. First and second columns represent a global image of the cell and a higher magnification view of the cell membrane, respectively.
