# Supplementary material for: Convergent Evolution of Calcineurin Pathway Roles in Thermotolerance and Virulence in Candida glabrata
Source: G3 (Bethesda). 2012 Jun 1;2(6):675–91. doi: 10.1534/g3.112.002279 (PMC3362297; doi:10.1534/g3.112.002279)
Supplement: Supporting Information [file supp_2.6.675_FigureS3.pdf]

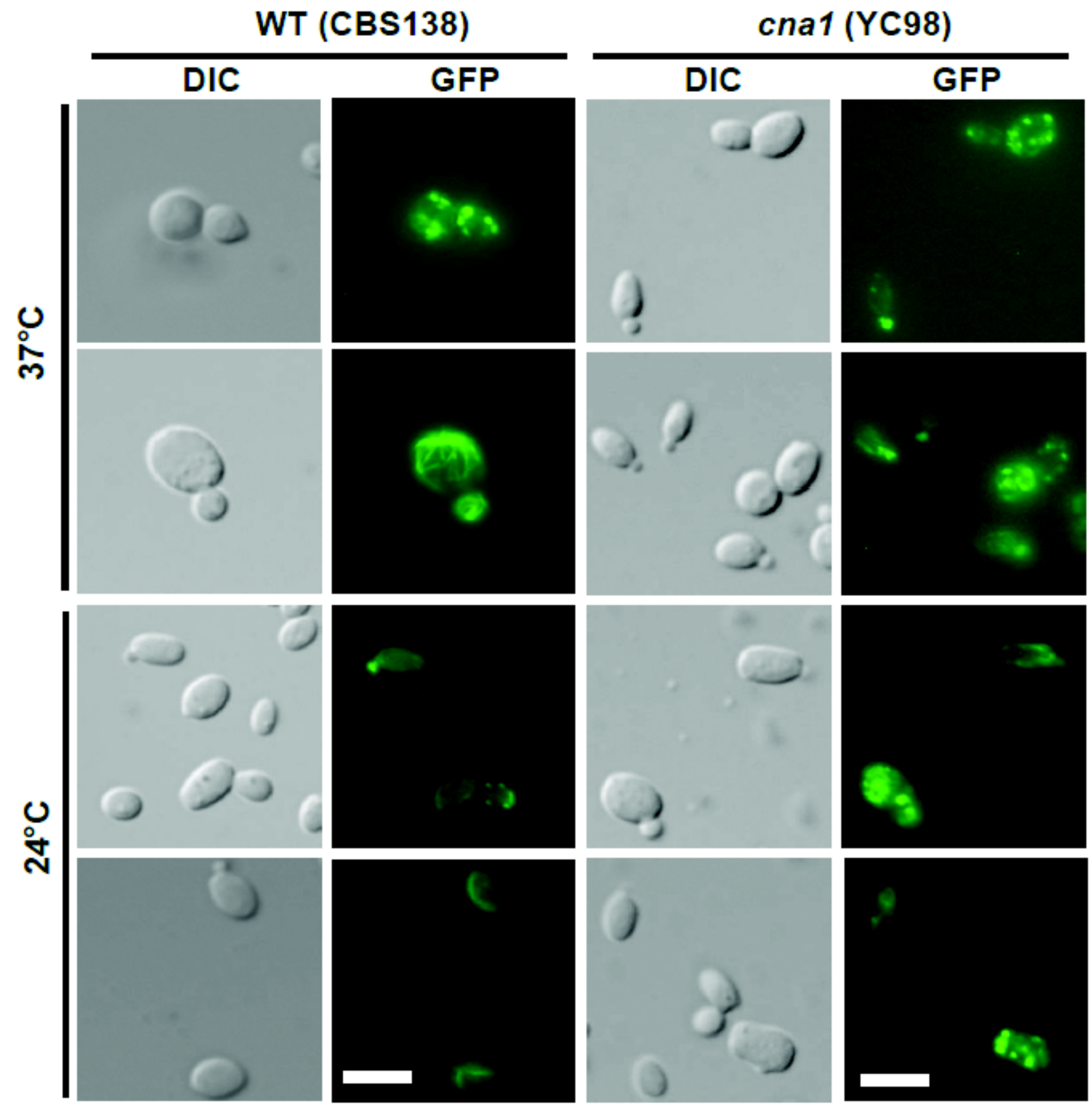

Figure S3 *C. glabrata* wild type and calcineurin mutant cells exhibit normal cortical actin patch structures. The incubation and staining of cells with Alexa fluor® 488 phalloidin are described in material and methods. The images were taken at 100X. Scale bar = 5 µm.
