# Supplementary material for: Convergent Evolution of Calcineurin Pathway Roles in Thermotolerance and Virulence in Candida glabrata
Source: G3 (Bethesda). 2012 Jun 1;2(6):675–91. doi: 10.1534/g3.112.002279 (PMC3362297; doi:10.1534/g3.112.002279)
Supplement: Supporting Information [file supp_2.6.675_FigureS4.pdf]

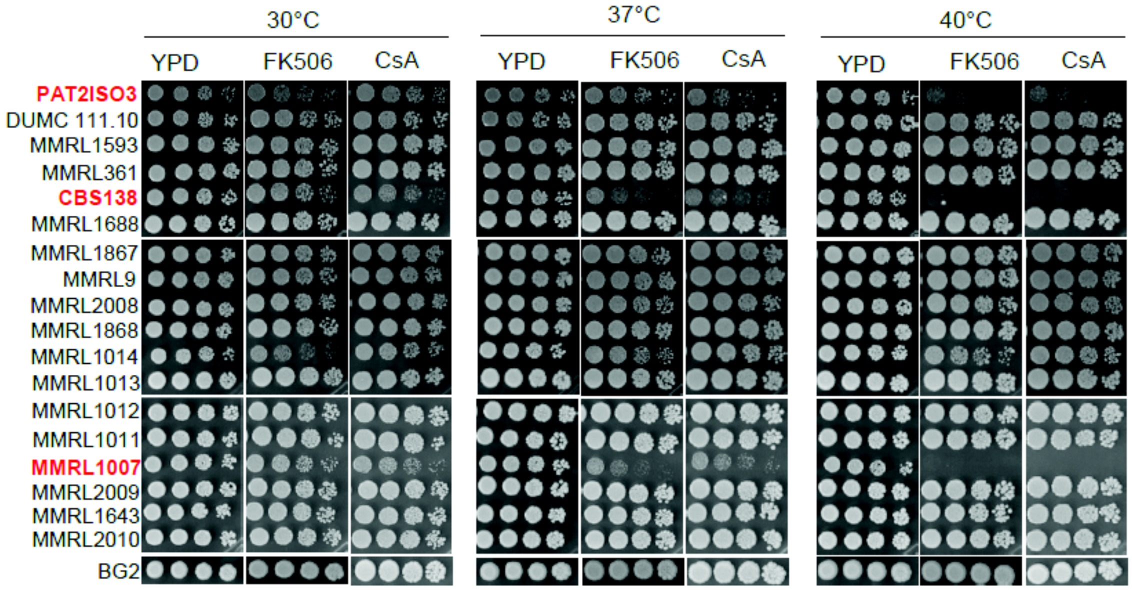

Figure S4 Some *C. glabrata* clinical isolates exhibit temperature-sensitive growth when exposed to calcineurin inhibitors. Cells were grown overnight in YPD at 30°C, 5-fold serially diluted, and spotted onto YPD medium containing FK506 (1 µg/ml) or cyclosporin A (CsA; 100 µg/ml). Cultures were incubated at the indicated temperatures for 48 h. *C. glabrata* strains that are hypersensitive to calcineurin inhibitors at high temperature are indicated in dark red.
