# Supplementary material for: Convergent Evolution of Calcineurin Pathway Roles in Thermotolerance and Virulence in Candida glabrata
Source: G3 (Bethesda). 2012 Jun 1;2(6):675–91. doi: 10.1534/g3.112.002279 (PMC3362297; doi:10.1534/g3.112.002279)
Supplement: Supporting Information [file supp_2.6.675_FigureS5.pdf]

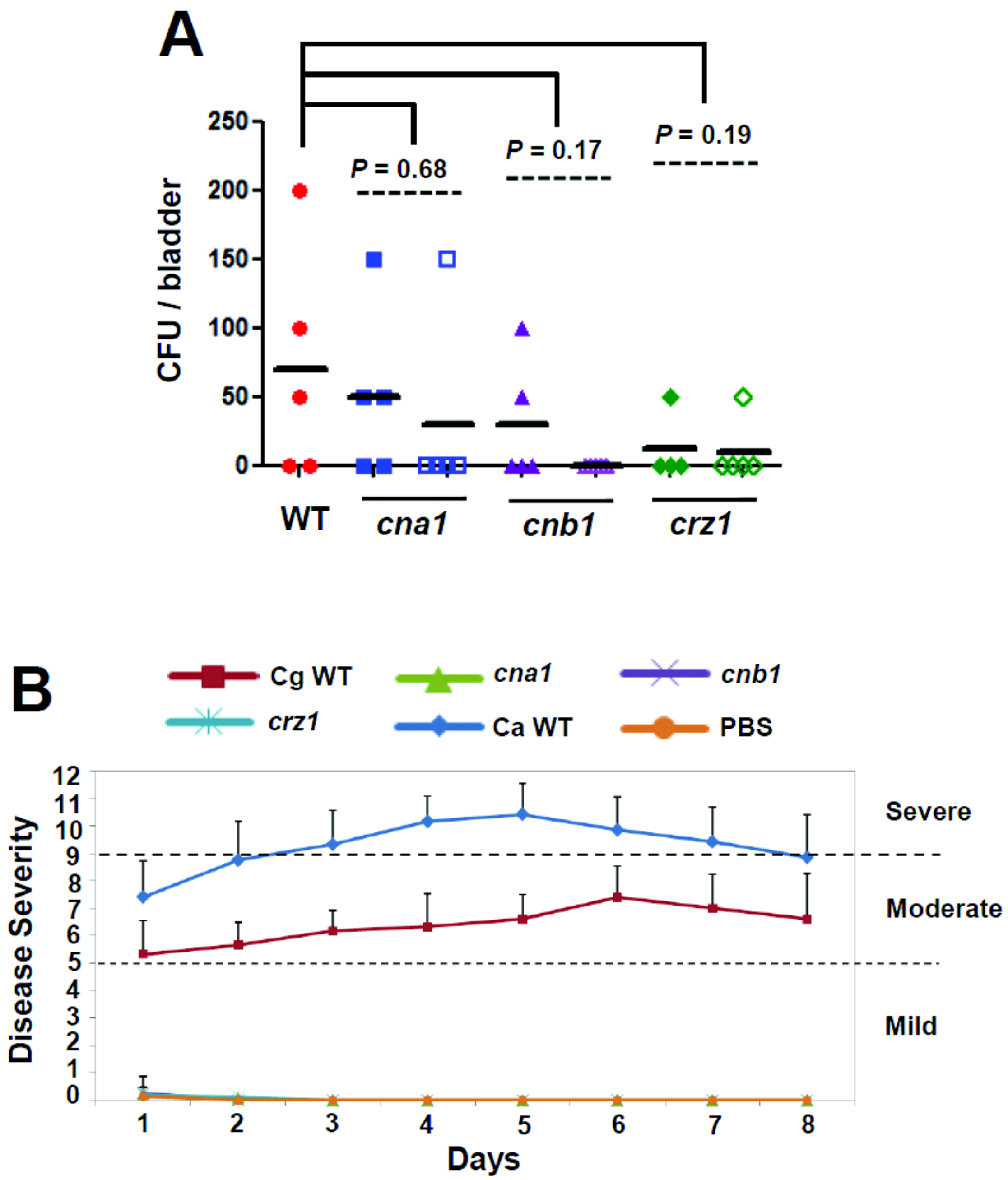

Figure S5 Virulence of *C. glabrata* calcineurin and *crz1* mutants in murine urinary tract and ocular infection models. **(A)** The fungal burden in the bladders was determined at day 7 after challenging mice with 3 x 10^7 cells via urinary tract infection. Five female C3H/HeJ mice were inoculated per strain (except one *crz1* mutant with 4 mice due to the death of one mouse following anesthesia treatment). The *P* value between wild-type (WT) and mutants is shown. **(B)** Disease severity was scored for 8 days. *C. albicans* SC5314 and the PBS mock inoculation served as reference controls. Mice infected with *C. glabrata cna1, cnb1,* or *crz1* mutants, or the PBS control, exhibited normal corneas, and score curves essentially overlapped. Mice infected with wild-type *C. glabrata* CBS138 and *C. albicans* SC5314 strains exhibiting visible signs of keratitis were plotted.
