# Supplementary material for: Convergent Evolution of Calcineurin Pathway Roles in Thermotolerance and Virulence in Candida glabrata
Source: G3 (Bethesda). 2012 Jun 1;2(6):675–91. doi: 10.1534/g3.112.002279 (PMC3362297; doi:10.1534/g3.112.002279)
Supplement: Supporting Information [file supp_2.6.675_FigureS6.pdf]

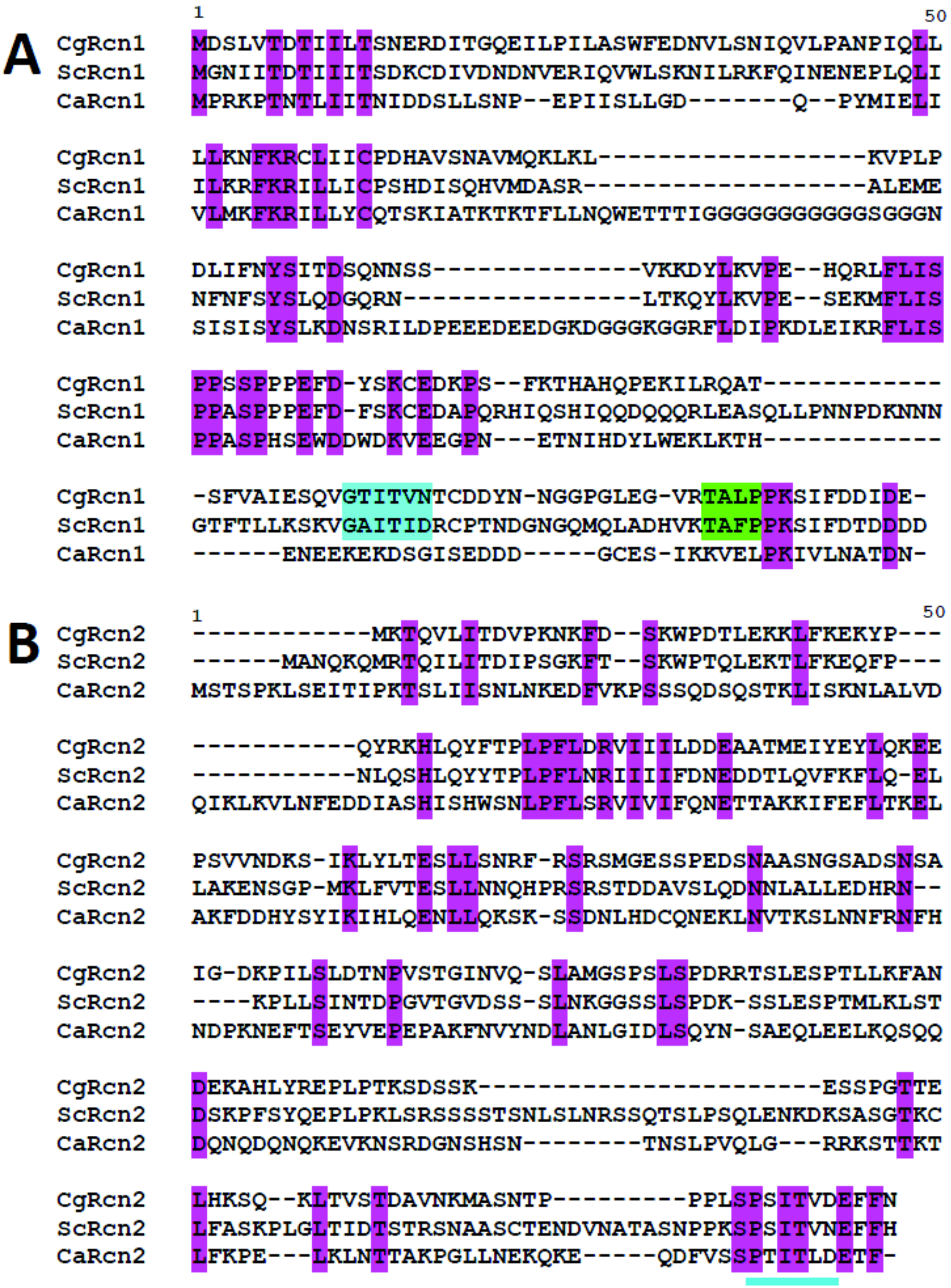

Figure S6 Pairwise alignment of calcineurin regulators from *C. glabrata*, *S. cerevisiae*, and *C. albicans*. Multiple sequence alignments are depicted using ClustalW software (http://npsa-pbil.ibcp.fr/cgi-bin/npsa_automat.pl?page=npsa_clustalw.html). The conserved amino acids and TxxP motif are purple- and green-shaded, respectively. The PxIxIT-like motif [P/G]x[I/V]x[I/V/L][E/D/N/H/T] is blue-shaded in panel A (depicting Rcn1 alignments), and underlined in panel B (depicting Rcn2 alignments).
