# Supplementary material for: Convergent Evolution of Calcineurin Pathway Roles in Thermotolerance and Virulence in Candida glabrata
Source: G3 (Bethesda). 2012 Jun 1;2(6):675–91. doi: 10.1534/g3.112.002279 (PMC3362297; doi:10.1534/g3.112.002279)
Supplement: Supporting Information [file supp_2.6.675_FigureS7.pdf]

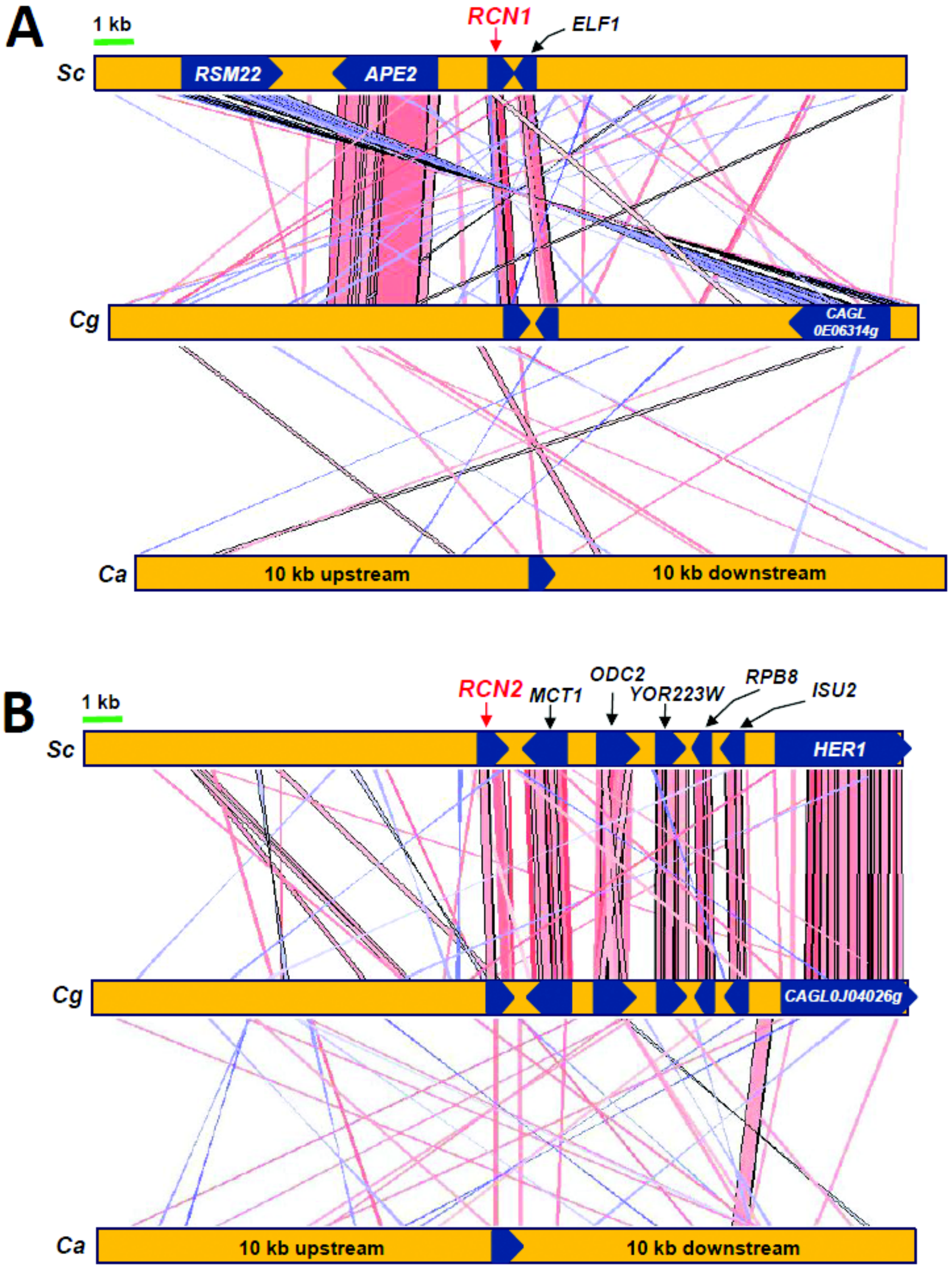


**Figure S7** Synteny analysis of Rcn1 and Rcn2. NCBI tBLASTx (Altschul *et al.*, 1997) and Artemis programs (Carver *et al.*, 2008) were used to conduct synteny analysis. Red and blue blocks (lines) represent forward and reverse matches of the DNA sequence (tBLASTx based) among *S. cerevisiae* (*Sc*), *C. glabrata* (*Cg*), and *C. albicans* (*Ca*).

**REFERENCES**

Altschul, S.F., Madden, T.L., Schaffer, A.A., Zhang, J., Zhang, Z., Miller, W., and Lipman, D.J. (1997) Gapped BLAST and PSI-BLAST: a new generation of protein database search programs. *Nucleic Acids Res* **25**: 3389-3402.

Carver, T., Berriman, M., Tivey, A., Patel, C., Bohme, U., Barrell, B.G., Parkhill, J., and Rajandream, M.A. (2008) Artemis and ACT: viewing, annotating and comparing sequences stored in a relational database. *Bioinformatics* **24**: 2672-2676.
