# Supplementary material for: Convergent Evolution of Calcineurin Pathway Roles in Thermotolerance and Virulence in Candida glabrata
Source: G3 (Bethesda). 2012 Jun 1;2(6):675–91. doi: 10.1534/g3.112.002279 (PMC3362297; doi:10.1534/g3.112.002279)
Supplement: Supporting Information [file supp_2.6.675_TableS1.pdf]

Table S1 PCR primers

Primer	Use	Sequence (5′ → 3′)
JC17	*SAT1* flipper	GGCCCCCCCTCGAGGAAGTT
JC18	*SAT1* flipper	GCTCTAGAACTAGTGGATCT
JC49	5′NCR of *CNA1*	ACACATTATGCAACATCAA
JC50	5′NCR of *CNA1*	AACTTCCTCGAGGGGGGGCCATTGTCGGTGCCAACAAGAA
JC51	3′NCR of *CNA1*	AGATCCACTAGTTCTAGAGCTTTCATCTCAAGAGCAAAA
JC52	3′NCR of *CNA1*	TTGAACGTTACTGGGGTTGA
JC55	*CNA1* ORF	AAGACAGACGCAGAGCTGAAT
JC56	*CNA1* ORF	GACATTCGTTTTTGAAAGTGA
JC48	Disruption confirmation	ACAATCAAAGGTGGTCCT
JC81	Disruption confirmation	AACTTCCTCGAGGGGGGGCC
JC122	Disruption confirmation	CACACTTTTATTGTTGTCGCC
JC134	5′NCR of *CNB1*	ACACCATAAAAAGTCCCAGCG
JC135	5′NCR of *CNB1*	AACTTCCTCGAGGGGGGGCCTTCCCTTGATTAATACTTCTC
JC136	3′NCR of *CNB1*	AGATCCACTAGTTCTAGAGCGGCAAGAAACTTAACGAGGA
JC137	3′NCR of *CNB1*	TCAACTTCAACAGAGTAGCTC
JC138	*CNB1* overlap	CAAACGTTTTGTTGGATGGC
JC139	*CNB1* overlap	CAACACTTTCGGAGAGAGAT
JC140	*CNB1* ORF	ATGGGAGCTGCACCATCTAAA
JC141	*CNB1* ORF	TACTGAAGGGTCAGGCTCTTT
JC142	5′NCR of *CRZ1*	TAAACCCATGGAGTGTGGAA
JC143	5′NCR of *CRZ1*	AACTTCCTCGAGGGGGGGCCGCTGAATATTGCAAAATCTTG
JC144	3′NCR of *CRZ1*	AGATCCACTAGTTCTAGAGCCACAAACCTCCAGTATTTTT
JC145	3′NCR of *CRZ1*	TCAAGTCTTAGAGATTCACCA
JC146	*CRZ1* overlap	TAATGAAAGCAATGCCAA
JC147	*CRZ1* overlap	GCAACAAATTTCTTGACTGGT
JC148	*CRZ1* ORF	TGGGCGATAACGAAGAGGATA
JC149	*CRZ1* ORF	CTCAAGTTATTTGAAGATGCA
JC441	5′NCR of *RCN2*	CCGTCGGGCTTCTTTAAAT
JC442	5′NCR of *RCN2*	AACTTCCTCGAGGGGGGGCCTTGTGCTGCTATATCTGCGTG
JC443	3′NCR of *RCN2*	AGATCCACTAGTTCTAGAGCGGGGATTGCTTCTATGAAGCT
JC444	3′NCR of *RCN2*	AAATTGCTCACGGAGTCACCT
JC445	*RCN2* overlap	CTCGGGATCATTGCTCAATA
JC446	*RCN2* overlap	AACGGGTCTAGTCGAGGACTT
JC453	*RCN2* ORF	AAAGGATCCATGAAGACACAGGTATTGA
JC454	*RCN2* ORF	AAAGGGCCCCTAGTTAAAGAACTCATCGAC
JC459	5′NCR of *RCN1*	CGAGTGTTTATTATTCGGG
JC460	5′NCR of *RCN1*	AACTTCCTCGAGGGGGGGCCTCCGATAAGAATGTTCAGATG
JC461	3′NCR of *RCN1*	AGATCCACTAGTTCTAGAGCGTACCTATCATGGTTGGTTAA
JC462	3′NCR of *RCN1*	TGTGATCTGCTCGATTTTACC
JC463	*RCN1* overlap	CCATTAACTATAGACGTTGCT
JC464	*RCN1* overlap	AACAGCACATAGAAAGATCAA
JC465	*RCN1* ORF	ACTCACGTCTAATGAAAGGGA
JC466	*RCN1* ORF	ATTTTGGTGGCAATGCCGTT
JC370	qPCR *ACT1* ORF	GTACCACCATGTTCCCAGGT
JC371	qPCR *ACT1* ORF	ACCACCGATCCAGACAGAGT
JC437	qPCR *CNA1* ORF	ACAGAGCGCTGTTCCTGATT
JC438	qPCR *CNA1* ORF	TGGTTGGGAAGTCCTGTTTT
JC439	qPCR *CRZ1* ORF	GGGCGATAACGAAGAGGATA

JC440	qPCR *CRZ1* ORF	TACATCGCCATTCATGCTGT
JC431	qPCR *YPS5* ORF	CAAGATCAGCGATGAACTAACG
JC432	qPCR *YPS5* ORF	TACTTGCGCTGACAAACCAC
JC435	qPCR *RCN2* ORF	CAGTACTTCACGCCGCTTC
JC436	qPCR *RCN2* ORF	CGATAACAGGGACTCCGTCA

Underline indicates sequences complementary to the *SAT1* flipper
