# Supplementary material for: Convergent Evolution of Calcineurin Pathway Roles in Thermotolerance and Virulence in Candida glabrata
Source: G3 (Bethesda). 2012 Jun 1;2(6):675–91. doi: 10.1534/g3.112.002279 (PMC3362297; doi:10.1534/g3.112.002279)
Supplement: Supporting Information [file supp_2.6.675_TableS2.pdf]

Table S2 Genes induced by FK506 but not by calcineurin mutation.

ORF	Gene	Fold change (FK506 / WT)	Functional description
CAGL0M12947g		10.2	Unknown
CAGL0M01760g	*PDR5*	9.5	Plasma membrane ATP-binding cassette multidrug transporter
CAGL0M09713		5.3	Unknown
CAGL0F02717g	*PDR15*	2.8	Plasma membrane ATP-binding cassette multidrug transporter
CAGL0E06688g		2.3	Unknown
CAGL0K09702g		2.2	Unknown
CAGL0C03223g	*SDH2*	1.9	Iron-sulfur protein subunit of succinate dehydrogese
CAGL0M09735g	*MEC3*	1.9	Damage and meiotic pachytene checkpoint protein
CAGL0M01870g		1.8	Unknown
CAGL0G00242g	*YOR1*	1.8	Plasma membrane ATP-binding cassette multidrug transporter
CAGL0C03289g	*YBT1*	1.6	Bile acid transporter
CAGL0K12958g		1.4	Unknown
CAGL0B01947g	*INO2*	1.3	Heteromeric Ino2/Ino4 basic helix-loop-helix transcription activator
CAGL0L09603g	*DSN1*	1.3	Important for chromosome segregation
CAGL0C04433g	*MCD1*	1.3	Required for sister chromatid cohesion in mitosis and meiosis
CAGL0I04180g	*CUP2*	0.8	Copper-binding transcription factor
CAGL0M09889g		0.7	Unknown
CAGL0J08184g	*ALP1*	0.7	Arginine transporter
CAGL0G06358g	*SNC1*	0.7	Vesicle membrane receptor protein
CAGL0A01089g		0.6	Unknown
